# Choline and Its Companions: Inter-Related Roles of Choline and B Vitamins in Fetal Development and Offspring Health

**DOI:** 10.3390/nu18142218

**Published:** 2026-07-08

**Authors:** Emma J. Derbyshire

**Affiliations:** Nutritional Insight, Surrey KT17 2AA, UK; emma@nutritional-insight.co.uk; Tel.: +44-(0)7584-375246

**Keywords:** conception, choline, fetal development, folate, neurodevelopment, nutrient synergy, pregnancy, vitamin B12

## Abstract

**Background/Objectives**: Previous publications have primarily examined the individual roles of nutrients during fetal development. However, growing evidence suggests that one-carbon (1C) metabolism nutrients, including choline and key B vitamins, act synergistically within interconnected metabolic pathways that modulate epigenetic regulation and may have implications for the health of future generations. **Methods:** This narrative integrative review examined evidence relating to the roles of choline and B vitamins (B1, B2, B6, folate (B9) and B12) in fetal development and offspring health. Peer-reviewed literature was identified through PubMed, Science Direct and Semantic Scholar. **Results:** Current evidence indicates that periconceptional and maternal intake and status of 1C metabolism nutrients are associated with DNA methylation processes involved in developmental programming and the risk of non-communicable diseases (NCDs) in childhood and adulthood. Habitual intakes of several 1C metabolism nutrients are frequently below recommended levels during pregnancy and lactation, particularly for choline and folate. Inadequate intakes, each contributing differently to 1C metabolism, may disrupt 1C metabolic pathways and alter DNA methylation patterns during critical windows of fetal programming. Homocysteine metabolism is intricately linked to 1C metabolism and is modulated by choline and B vitamins. Collectively, these pathways have potential implications for the health of the next generation, including effects on growth, neural tube closure, brain development and increased susceptibility to diseases later in life, e.g., cardiovascular disease, diabetes, obesity and other chronic conditions. Adequate maternal intakes of choline and B vitamins may help mitigate the ‘early life origin’ of certain NCDs by promoting healthy neurodevelopment, reducing inflammation, and regulating central metabolic pathways. **Conclusions:** Greater awareness of the roles and importance of 1C metabolism nutrients, including choline and key B vitamins (B1, B2, B6, folate and B12), during the early life course is warranted. Furthermore, there is also a need for organizations and policy makers to formalize intake recommendations for 1C metabolism nutrients beyond the individualized simplicity of folate/folic acid, and to extend this to include other methyl-donor nutrients with epigenetic effects, such as choline and key B vitamins, given their interconnected roles in 1C metabolism and fetal development.

## 1. Introduction

Nutrition is one of the most important environmental factors that can influence early development through the regulation of epigenetic mechanisms (biological changes that control how genes are switched on or off without altering the DNA sequence) [1,2]. Women who are healthy upon the time of conception are more likely to have a healthy pregnancy and child [3]. An adequate supply of nutrients during this time is a critical determinant of fetal outcomes, as essential nutrients are needed for growth and healthy development [4,5,6]. Methyl-donor nutrients such as choline and key B vitamins (riboflavin (B2), pyridoxine (B6), folate (B9) and cobalamin (B12)) can modulate epigenetic regulation, with important implications for optimal fetal development and long-term offspring health [7,8].

There is now a plethora of evidence that maternal nutrition during the first 1000 days of the offspring’s life can profoundly influence fetal programming through maternal–fetal–placental interactions [9,10,11]. From a historical perspective, the British epidemiologist David Barker was one of the first scientists to develop the “thrifty phenotype” hypothesis and suggest that poor fetal/infant growth was associated with metabolic disease, e.g., type 2 diabetes later in adulthood, with a nutrient-depleted intrauterine environment thought to have permanent underpinning effects on fetal body structure, function and metabolism [12,13,14,15,16,17,18]. Imbalances or deficiencies of key nutrients during the early life course can alter epigenetic programming, with effects potentially persisting into adulthood; a concept now more widely referred to as the Developmental Origins of Health and Disease (DOHaD) hypothesis [1,19,20,21]. The DOHaD theory proposes that environmental exposures during critical periods, from conception through to childhood, can permanently program long-term health, increasing the risk of neurodevelopmental alterations [19] and vulnerability to chronic diseases such as obesity, diabetes, cardiovascular disease and certain cancers [1,11,20,21,22,23].

Early-life influences, including nutrition, stress, infections, and toxins can all significantly alter gene expression through epigenetic mechanisms creating “predictive adaptive responses” that favour survival in harsh environments but can lead to diseases if the environment later changes, creating a “developmental mismatch” [24,25]. From a nutrition perspective, healthy and balanced diets are important providers of methyl-donating nutrients, such as choline and B vitamins [8,26]. However, highly refined and processed diets and those excluding animal-derived foods can be lower in some of these micronutrients [27,28,29,30,31]. Furthermore, food deprivation arising from factors such as political instability, climate-related events, rising food costs, limited nutrition education, and inadequate food storage conditions has been associated with micronutrient deficiencies that could compromise nutrient-dependent metabolic processes that are essential for growth, development, and long-term health outcomes [32,33,34].

Previous research has largely focused on the individual effects of specific methyl-donor nutrients; however, few studies have examined the combined roles of choline and key B vitamins in 1C metabolism during periods of fetal development [8,33]. Considering this, the present narrative integrative review [35] analyzes and synthesizes evidence from published studies on the epigenetic and metabolic roles of choline and key B vitamins, reviews current habitual intakes and dietary recommendations, and examines their implications for fetal development and offspring health.

## 2. Methyl-Donor Nutrients in Fetal Development and Offspring Health

1C metabolism comprises a network of interconnected pathways that generate and transfer 1C methyl (–CH_3_) units required for fetal development, providing a key mechanistic link between nutrition and epigenetic regulation [36,37]. The pathways support the transfer of 1C units for nucleotide biosynthesis and methylation reactions involving DNA, RNA, and proteins [37]. Methyl groups and nucleotides are required for DNA synthesis, repair, and gene expression regulation, making 1C metabolism critical for cell growth and function across all tissues [37].

Several nutrients act as cofactors, methyl donors, or methyl acceptors within 1C metabolism (Table 1) [38]. These include choline and B vitamins (B2, B6, folate (B9) and B12), which are central to 1C transfer pathways and influence epigenetic processes including DNA methylation (biological tags that influence gene activity), histone modification (changes to DNA-packaging proteins that control how easily genes can be accessed) and microRNA expression (small molecules that help fine-tune gene expression) during critical developmental windows [1,14,19,39].

De novo synthesis of nucleotides requires both ribose-5-phosphate (R5P) and folate-derived 1C units for the formation of purine nucleobases and thymidylate [43,44]. Although vitamin B1 (thiamine) is not directly involved in the phosphate pathway, it serves as an essential cofactor in the generation of R5P through the pentose pathway [43,44,47] and assists glucose metabolism, cellular energy production, and DNA repair processes [46]. Given these complementary functions, thiamine is considered within the broader context of pathways reinforcing fetal growth and development.

**Figure 1:**
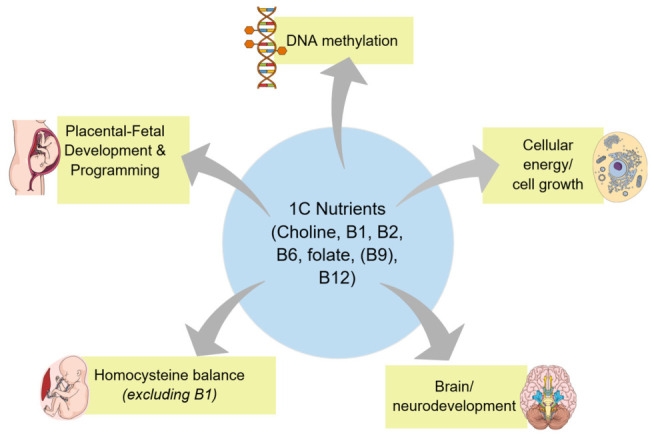
Importance of 1C nutrients and vitamin B1 in fetal development and offspring health. References: DNA methylation [23,53,56]; cellular energy/cell growth [46]; brain/neurodevelopment [19,55,62]; homocysteine balance (excluding B1) [58,59,61]; placental–fetal development and programming [16,19,50,63].

Collectively, these pathways help maintain the fetal epigenetic landscape by supplying methyl groups required for DNA methylation, with disruptions potentially affecting fetal/neonatal metabolic programming and placental development [23,53]. The principal effects of choline and B vitamins on fetal development and offspring health are shown in Figure 1. Methyl-donor nutrients are important for nervous system and brain development during pregnancy [54]. Epigenetic alterations may modify patterns of gene expression and increase susceptibility to neurodevelopmental, metabolic, or other chronic health issues later in life, positioning epigenetic mechanisms as a biological bridge “from womb to mind” [55,56,57]. Inadequate intakes of B6, B9 (folate), B12 and choline have also been associated with hyperhomocysteinemia [58,59,60], which has been linked to a range of adverse pregnancy outcomes (Section 5.3) [59,61].

Overall, several key epigenetic mechanisms regulating gene expression are nutrient responsive, particularly to methyl-donor nutrients, emphasizing the importance of adequate nutrition during fetal development [19,33,34].

## 3. Nutrient Requirements and Current Intake Gaps

### 3.1. Nutrient Requirements

The European Food Safety Authority (EFSA) has developed average requirement (AR)/adequate intake (AI)/population reference intake (PRI) recommendations for folate, vitamins B1, B2, B6 and adequate intakes (AIs) for choline and vitamin B12 (Table 2). The AR is defined as “*the level of a nutrient in the diet that meets the daily needs of half of the people in a typical healthy population*” whilst the AI is “*the average nutrient level consumed daily by a typical healthy population that is assumed to be adequate for the population’s needs*” which is used when there is not enough data to derive an AR [64]. The PRI is “*the intake level that is adequate for almost all healthy people in a population group*” [64].

Nutritional requirements are considerably different in pregnancy and breast-feeding when compared to those of non-pregnant women due to a series of physiological changes which include maternal and fetal uptake, placental accretion and losses through breast milk [65,66,67,68,69,70,71,72]. For pregnancy, an AI of 480 mg/day of choline has been derived by extrapolating the AI from non-pregnant women and accounting for gestational weight gain whilst the AI of 520 mg choline/day for lactating women is based on non-lactating requirements and estimated choline losses in breast milk (≈120 mg/day; when exclusively breastfeeding) [72]. In pregnancy maternal hepatic synthesis of choline increases but choline demands during pregnancy appear to exceed current intake recommendations [66].

Choline can be synthesized de novo via methylation of phosphatidylethanolamine, a reaction catalyzed by phosphatidylethanolamine N-methyltransferase (PEMT) [73]. The PEMT pathway catalyzes endogenous choline synthesis via estrogen-responsive methylation of phosphatidylethanolamine and is upregulated during pregnancy; however, inter-individual variability due to common PEMT polymorphisms can reduce endogenous synthesis capacity and increase dietary choline requirements [74,75]. More than 98 polymorphisms have been identified in PEMT, indicating these are common rather than rare [76].

### 3.2. Intakes and Deficiencies

Several key studies have measured habitual dietary intakes of these nutrients during pregnancy (Table 2). Firstly, focusing on choline, Derbyshire et al. (2021) systematically reported intakes ranging from 233 mg/day to 383 mg/day for women across the childbearing years; even with supplements intakes fell below the recommended intakes of 480 mg/day and 520 mg/day for pregnancy and lactation, respectively [72,77,78,79]. In Spanish pregnant women (n = 133) mean intakes were 271 mg/day for choline, 1.4 mg/day for vitamin B6, 182.8 μg/day for folate and 4.5 μg/day for vitamin B12 [80]. Subsequently, intakes were generally adequate for vitamin B12 but vitamin B6, folate and choline were under-consumed [80]. Mean intakes from another Spanish study—the ECLIPSES study—are also reported in Table 2. The daily intake as a percentage compared to the Recommended Dietary Allowances (RDA) for all three trimesters was 66% for vitamin B1, 95% for B2, 68% for B6, 34% for folate and 171% for B12, indicating that intakes for B vitamins were mostly insufficient except for B12 [81]. In the UK, folate was consumed in very low amounts by Scottish expectant mothers (237–323 μg/day) [82].

Regarding prevalence of deficiencies, it is important to consider that different studies define this differently. Folate deficiency is typically diagnosed via whole blood/erythrocyte folate rather than serum/plasma folate [83]. In the UK the prevalence of serum folate and vitamin B12 deficiency has been reported to be 12.4% and 6.4%, respectively [84]. Findings from the latest UK National Diet and Nutrition Survey which referred to red blood cell folate levels < 748 nmol/L as a marker of deficiency reported that 83% women of childbearing age (16 to 49 years) were deficient [85]. In Dublin, Ireland, the ROLO study (n = 759 mother–child pairs) reported that dietary folate intakes were deficient in all three trimesters (mean intakes 253, 265 and 259 µg/day; 89.6–91.1% deficient) whilst mean vitamin B12 intakes were 3.8, 4.1 and 4.2 µg/day in the first, second and third trimesters, respectively (2.9–5.3% deficient) [86].

**Table 2:** EFSA nutrient requirements and range of average daily intakes for 1C metabolism nutrients.

	Non-Pregnant Females	Pregnancy	Lactation	Range of Average Daily Intakes
AI for Choline (mg/day) [72]	400	480	520	**233–383** [72,77,78,79]
PRI B1 (mg/MJ and mg/d) [68]	0.1 and ≈0.9	+0.03, +0.11, +0.21 *	+0.21 *	0.9–2.2 [81,82]
PRI B2 (mg/d) [69]	1.6	1.9	2	1.3–2.0 [81,82]
PRI B6 (mg/d) [67]	1.6	1.8	1.7	1.3–2.4 [80,81,82]
PRI for Folate (μg DFE/day) [70]	330	600	500	**183**–**323** [80,81,82,86]
AI for Vitamin B12 (μg/day) [71]	4	4.5	5	3.8–5.1 [80,81,82,84,86]

Key: AI, adequate intake; DFE, dietary folate equivalents (folate bioavailability measure); PRI, population reference intakes. * EFSA’s DRV 2016 Appendix K [68] and presented for the 1st, 2nd, and 3rd trimester and lactation, respectively. Bold text means that nutritional shortfalls are evident.

The rate at which deficiencies develop also differs substantially among 1C metabolism nutrients. Vitamin B12 deficiency may take years to manifest in well-nourished adults because of substantial hepatic stores, whereas folate and thiamine deficiencies can develop within weeks to months due to limited body reserves [87,88,89]. In contrast, choline is not stored in a dedicated reserve pool and relies on continuous turnover within phosphatidylcholine-containing membranes and lipoproteins, making adequate dietary supplies particularly important during periods of rapid growth and development [90].

Overall, these data indicate that choline intake is generally suboptimal amongst women of childbearing age. Suboptimal folate intakes and high prevalences of deficiency are also apparent across European cohorts. Spanish data shows vitamin B1, B2 and B6 intakes often fall below the RDA [81]. Vitamin B12 intakes are generally adequate although up to 1 in 20 Irish pregnant women have been reported to have deficient intakes [86]. On a final note, it is also important to consider that mean intake data may obscure nutrient inadequacies and separate analyses of sub-groups, such as vegetarians and vegans, are frequently lacking.

## 4. 1C Nutrients and Epigenetic Gene Regulation

**Figure 2:**
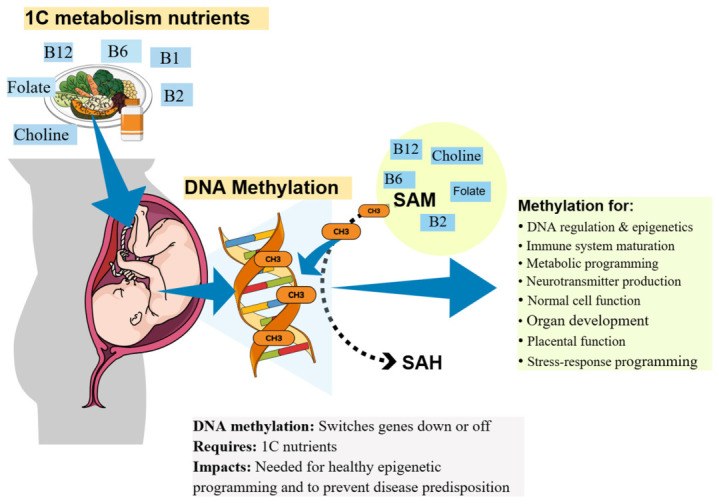
Epigenetic processes underpinning DNA methylation. Source: Adapted from Dusa et al. (2025) [97] and Ghazi et al. (2020) [112].

**Figure 3:**
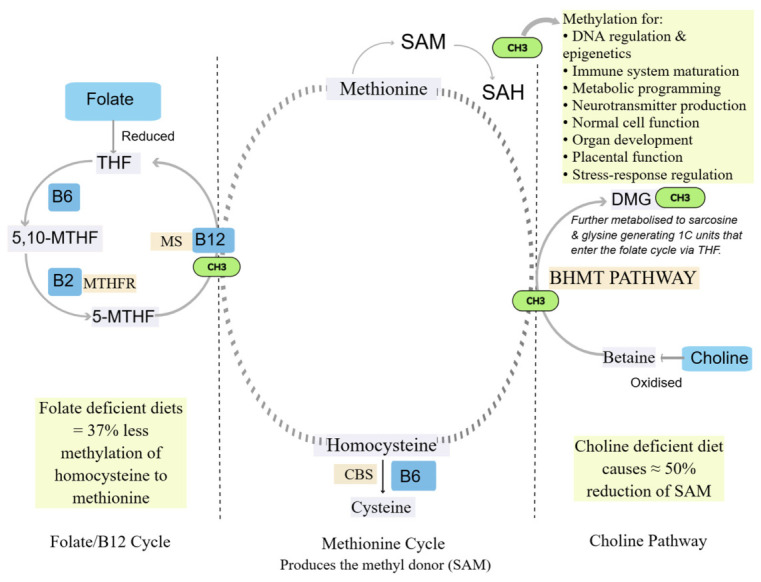
How choline, folate and B12 sustain methylation for fetal and child development across the early life course. Abbreviations: BHMT, betaine homocysteine methyltransferase enzyme; CBS, cystathionine-β-synthase enzyme; 5-MTHFR, 5-methyltetrahydrofolate; MTHFR, methyl-tetrahydrofolate reductase enzyme; MS, methionine synthase enzyme; SAH, S-adenosylhomocysteine; SAM, S-adenosylmethionine; THF, tetrahydrofolate. Adapted from: Bortz & Obeid (2025) [113], Steane et al. (2023) [114] and Randunu & Bertolo (2020) [7].

Fetal DNA methylation during pregnancy is a crucial, natural epigenetic process in which methyl groups are added to DNA to regulate gene expression [91,92]. Epigenetics is the study of heritable changes in gene expression and genome function that occur without altering the DNA sequence [93]. Defective DNA methylation, which regulates tissue-specific gene expression, can induce long-term changes in gene function and metabolism (Figure 2) [92,94,95]. Methyl groups for DNA methylation are largely derived from the diet and supplied through 1C metabolism of key nutrients [96,97]. As described later in the publication, choline, B2, B6, folate and B12 play critical roles in S-adenosylmethionine (SAM) synthesis (Figure 3), thereby increasing the availability of methyl groups for epigenetic regulation.

Interestingly, around 60 years after the Dutch Hunger Winter famine of 1944–1945 researchers began look at DNA methylation levels in those whose mothers had been pregnant during the famine [98]. They found that they had increased levels of DNA methylation in some genes and reduced DNA methylation levels in others, when compared to their siblings who had not been exposed to famine *in utero*, with these differences potentially explaining variations in disease risk later in life [99,100,101]. This is of importance because the individuals exposed to famine when their mother was carrying them in the womb had an increased risk of developing certain diseases, including heart disease, type 2 diabetes and schizophrenia [102]. Similarly, maternal intake of 1C metabolism nutrients, including vitamin B12, folate, and choline, has been associated with altered DNA methylation of metabolic genes in offspring, supporting a role for maternal methyl-donor status in early-life epigenetic programming [103].

Alongside this, emerging evidence has linked variants in the PEMT and methylenetetrahydrofolate (MTHFR) genes with nonalcoholic fatty liver disease (NAFLD; now called metabolic dysfunction-associated steatotic liver disease; MASLD) [104,105]. Future Mendelian randomization studies could help determine whether disruptions in choline and 1C metabolism play a causal role in NAFLD/MASLD pathogenesis rather than merely representing correlated risk factors. Among individuals carrying the MTHFR C677T variant, pre-pregnancy folic acid intake appears to have a greater impact on placental DNA methylation [106]. The MTHFR C677T polymorphism is an important determinant of 1C metabolism and has been associated with altered newborn DNA methylation profiles, likely through reduced methyl-group availability [36].

Furthermore, the MTRR 66A>G polymorphism, which alters the activity of the vitamin B12-dependent methionine synthase reductase enzyme, has been associated with an increased risk of spina bifida [107]. Polymorphisms in the cystathionine-β-synthase (CBS) gene may also alter activity of this vitamin B6-dependent enzyme, which catalyzes the conversion of homocysteine to cystathionine within the transsulfuration pathway and contributes to cysteine synthesis [108,109].

Overall, genetic polymorphisms in enzymes involved in 1C metabolism—including those depending on vitamin B1, B2, B6, folate (B9), B12 and choline as cofactors—can reduce pathway efficiency which may result in decreased methyl-group availability, impaired DNA methylation and elevated homocysteine levels [110,111]. Consequently, individuals carrying such variants may have increased choline and B vitamin requirements and through their effects on methylation-dependent processes, these genetic differences may influence fetal epigenetic programming, with potential downstream consequences for brain development, fetal growth, cardiometabolic regulation and other long-term health outcomes [19,97].

## 5. Interdependent Roles of Choline and 1C-Related B Vitamins

### 5.1. Metabolic Interactions

The childbearing years are a life stage when methyl-donor supply is crucial and demand for methyl-donor nutrients is even higher due to physiological demands [66,115,116]. 1C metabolism involves a tightly interconnected network of pathways in which choline, vitamin B2, B6, folate and vitamin B12 work together to fuel production, transfer and utilization of 1C units [47,117,118]. Methyl-groups are supplied via two main processes: (1) the diet (dietary methyl donors) or (2) generated endogenously through 1C metabolism via methyl neogenesis (the formation of new methyl groups), primarily through the formation of SAM, the principal cellular methyl donor [113]. Methyl groups are required for numerous cellular processes, including DNA synthesis and methylation, regulation of gene expression, amino acid and protein metabolism, hepatic phosphatidylcholine synthesis via the PEMT pathway for very low-density lipoprotein production and lipid export, and glutathione production for antioxidant defence [47,119]. Methyl group metabolism predominantly takes place in the liver and muscle organs [119].

As shown in Figure 3, choline and vitamins B2, B6, folate (B9) and B12 are tightly interconnected within 1C metabolism, where they reinforce methylation reactions that generate SAM, the universal methyl donor, as well as DNA regulation and epigenetic mechanisms [113,120]. Deficiency in one nutrient increases reliance on the others, forming a compensatory metabolic network that maintains cellular methylation and homocysteine recycling essential for normal fetal development and function [26,47,113,121].

Approximately half of all cellular SAM is provided by the betaine homocysteine methyltransferase (BHMT) pathway, and a choline-deficient diet can reduce SAM production by 50% and liver folate by 31–40%; Figure 3 [113]. Choline contributes to 1C metabolism through its oxidation to betaine, which serves as a methyl donor for the remethylation of homocysteine to methionine via the BHMT pathway, generating dimethylglycine (DMG) [120,122]. DMG is then further metabolized to sarcosine and then glycine, with these reactions transferring 1C units to tetrahydrofolate (THF), thereby linking choline-derived methyl groups to the folate-mediated 1C network [51,113]. This helps to regulate intracellular pools of SAM and S-adenosylhomocysteine (SAH) thereby influencing the availability of methyl groups for epigenetic processes such as DNA methylation which, in turn, regulates gene expression [40,120].

Alongside this, folate and vitamins B2, B6 and B12 (blue boxes; Figure 3) are central to the folate–methionine cycle [47,121,123]. Folate is first reduced to tetrahydrofolate (THF) and then converted to 5,10-methylene THF (5,10-MTHF), an enzymatic step that requires vitamin B6 as a cofactor [26,47,121,124]. 5,10-MTHF is then converted to 5-methyltetrahydrofolate (5-MTHF) by the enzyme methylenetetrahydrofolate reductase (MTHFR) to which vitamin B2 is an active cofactor [26,118]. B2 deficiency can impair this reaction and impede methyl-donor generation for methylation reactions [118]. 5-MTHFR then donates methyl groups to homocysteine for the conversion to methionine in a reaction catalyzed by methionine synthase (MS) which requires vitamin B12 as a cofactor [47,121,125]. This regenerates methionine, which is then used to form SAM which is a universal methyl donor for different methyltransferases present in cells [47,121,126]. Lastly, the conversion of homocysteine into cysteine also requires vitamin B6-dependent enzymes as cofactors [127,128].

In general, it is also important to consider that choline and folate have an interdependent metabolic relationship, such that deficiency in one can disrupt the metabolism of the other [129]. Likewise, vitamin B12 deficiency can also contribute to functional folate deficiencies, as 5-MTHF in an irreversible reaction is converted by methionine synthase into THF which is a vitamin B12-dependent reaction [26].

Taken together, both remethylation pathways rely on choline-derived methyl-groups. Betaine directly supports homocysteine remethylation to methionine via BHMT, while its metabolite dimethylglycine contributes 1C units to the folate cycle, thereby supporting the folate- and vitamin B12-dependent methionine synthase pathway [51,113]. Choline and B2, B6, folate (B9) and B12 help to sustain adequate methyl-donor pools, particularly SAM which is crucial for efficient methylation reactions involved in DNA regulation, epigenetic programming, organ development, placental function, metabolic health programming, neuroendocrine stress-response regulation and others [47,113,114,117,120,130,131]. Dietary shortfalls may impair the activity of enzymes involved in these cycles, thereby reducing methyl group availability for DNA synthesis and epigenetic regulation which may in turn affect fetal development and offspring long-term health [47,118,125].

### 5.2. Epigenetic Programming in Pregnancy

The first 1000 days of life (from preconception to two years of age) constitute a critical period in which early nutrition influences developmental programming via epigenetic mechanisms, affecting the risk of cardiovascular disease, diabetes, obesity, and other chronic conditions later in life [11,132,133,134]. This critical life stage has been referred to as “*the neonatal window of opportunity–early priming for life*” [135]. Developmental plasticity, defined as the capacity to adapt during development, extends from preconception through early childhood and involves epigenetic responses to environmental influences, including nutrient supply [136]. Therefore, early nutrition has the potential to exert significant, lifelong health effects, as epigenetic changes during ‘in utero’ development can create lasting biological ‘memories” [137]. Furthermore, other factors, such as maternal stress, infection and environmental exposures (e.g., smoking, alcohol, heavy metals, pollutants) can also affect the epigenome [55,138].

**Figure 4:**
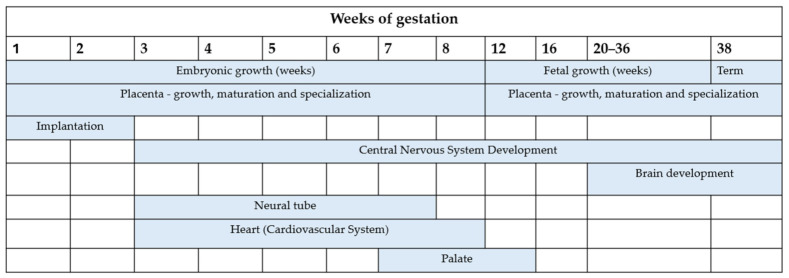
Organ system development and placental growth, maturation and specialization influenced by 1C nutrients. Abbreviations: DHA, docosahexaenoic acid. Source: Adapted from: Derbyshire (2011) [142]; Selevan et al. (2000) [143].

Maternal nutrient supply at different critical windows of gestation coincide with (1) embryogenesis, (2) placental growth and (3) fetal growth (Figure 4) [139]. Embryogenesis generally occurs during the first 8 weeks of pregnancy; a process when all of the organ systems begin to form from germ cell layers [140]. This period is particularly important as this is when the embryo is vulnerable to the effects of poor maternal nutrition, or other stressors which can affect structural development [141]. The central nervous system is one of the first organ systems to develop, followed by the cardiovascular system, with the palate developing slightly later [142,143]. There is emerging evidence that ‘trimester-specific’ nutrients can impact on sensitive windows of epigenetic programming having lasting effects into childhood [144]. In essence, physiological changes in pregnancy that contribute to a decline in B vitamin status add another further layer of complexity to the regulation of 1C metabolism [52]. From a developmental perspective, folic acid is particularly important for women of childbearing age before conception and early in the first trimester of pregnancy as this is a significant time for neural tube closure (around 4weeks post conception) [145,146,147]. Folic acid supplementation from the start of pregnancy can also lower cleft lip and palate [148].

Folic acid is also required for placental growth and function throughout gestation [58,149,150]. A growing body of evidence suggests that maternal folate and related B vitamins modulate 1C nutrient metabolism and may shape offspring cognitive development through folate-dependent DNA methylation of brain-related genes, with potential implications for cognitive health later in life [151,152]. Furthermore, vitamin B1 is involved in neurotransmitter and myelin synthesis and plays a critical role in cellular energy production [46,153].

Evidence further suggests that low choline and vitamin B12 intakes during these developmental phases are associated with a higher risk of neural tube defects (NTDs) [117,118,154,155,156]. Choline serves as a crucial methyl donor in 1C metabolism, significantly influencing DNA and histone methylation, thereby altering gene expression related to fetal growth, brain development, stress responses, and long-term metabolic health [120,157,158]. Consequently, inadequate choline availability may result in adverse epigenetic changes with potential lifelong effects [111].

Choline is also important for brain development, acetylcholine synthesis (a neurotransmitter that is also stored in the placenta), phospholipid (phosphatidylcholine) synthesis for cell membrane formation [41,159,160], bile and lipoprotein secretion [161] and sphingomyelin production, which requires phosphatidylcholine-derived choline for the production of ceramides [162]. In turn, choline availability has been shown to influence neurodevelopmental gene expression [163] and infant information-processing speed during early infancy [164].

Finally, it should also be noted that choline and DHA have synergistic roles in fetal brain and eye development, alongside arachidonic acid, which is also critical for neurodevelopment, yet intakes of these nutrients often remain below recommended levels [165,166]. Earlier research suggests maternal physiological adaptations selectively enrich circulating DHA-containing phosphatidylcholine for fetal DHA supply, while fetal acquisition of polyunsaturated fatty acids is largely dependent on maternal lipid supply [167,168]. Choline supplementation in preterm infants has also been shown to enhance DHA incorporation into phosphatidylcholine, approximately doubling the increase achieved with DHA alone, which may improve DHA availability to the brain and eyes [169].

### 5.3. Homocysteine Metabolism and Regulation

Hyperhomocysteinemia has been reported in 21.2% of women of childbearing age in populations with high rates of folate and vitamin B12 deficiency, potentially impairing methylation reactions and adversely affecting reproductive outcomes and fetal development [84]. Homocysteine is a sulfur-containing amino acid that lies at the intersection of the folate, methionine, and glutathione pathways [170]. Choline and vitamins B2, B6, folate (B9) and B12 are functionally inter-related, acting as interdependent cofactors in homocysteine metabolism (Figure 3) [129,171,172]. Homocysteine is formed from methionine as an intermediate in 1C metabolism and metabolized through vitamin B6- and vitamin B12-dependent pathways [26]. Choline also plays an important role in homocysteine metabolism. Following its conversion to betaine, choline donates a methyl group to homocysteine to form methionine, generating dimethylglycine as a by-product, which is subsequently metabolized to sarcosine and glycine, contributing 1C units to the folate cycle (Figure 3) [113,173].

Supplementation of B vitamins has been found to reduce plasma homocysteine levels [170,174]. Remethylation of homocysteine to methionine requires 5-MTHF as the methyl donor and vitamin B12 as an essential cofactor for methionine synthase [121]. Methionine is then converted to SAM (the body’s primary and universal methyl donor for most methylation reactions) and later to SAH which is then cleaved to form homocysteine, with its metabolic fate dependent on B vitamin status [175]. Homocysteine breakdown to cysteine also occurs via the transsulfuration pathway which requires vitamin B6 [176].

Given these metabolic roles, it is well recognized that B vitamin inadequacies can result in elevated plasma homocysteine concentrations and hyperhomocysteinemia [177,178,179]. Elevated homocysteine levels are a well-studied indicator of disturbances in C1-metabolism and have consistently been associated with adverse pregnancy outcomes [180,181]. This includes NTDs [182,183], fetal growth restriction [58,184], pre-eclampsia [58], recurrent pregnancy loss [58], spontaneous miscarriage, preterm birth [58], and placental abruption [58,185].

## 6. Pregnancy and Developmental Outcomes

### 6.1. Neural Tube Development

Intakes of 1C nutrients can affect fetal development and future health. NTDs arise from the failure of neural tube formation during early embryonic development and are severe congenital malformations affecting around 1–2 per 1000 pregnancies [186]. Other publications report the European NTD prevalence range and median to be 1.3–35.9; 9.0 per 10,000 births whilst Eastern Mediterranean rates have been reported to be even higher at 2.1–124.1; 21.9 per 10,000 births [187]. Variations in prenatal detection, selective termination of pregnancies and stillbirths affected by NTDs can complicate epidemiological understanding of NTDs [188]. For example, within Europe, Ireland (8.74–9.58 NTD per 10,000 births) and Great Britain (12.14–15.08 NTDs per 10,000 biths) have consistently higher rates of NTDs, and within these islands there are higher prevalences at birth in the North and West [189].

Folic acid supplementation is known to reduce approximately 70% of all NTDs [190,191]. DNA hypomethylation can cause abnormal microRNA (miRNA) expression during neural development, disrupting neural gene regulation and contributing to the spectrum of phenotypes observed in NTDs [190,192]. miRNAs are key regulators involved in central nervous system development, regulating gene expression related to mechanisms behind NTDs [193]. Abnormal miRNA expression is associated with the disruption of critical processes such as apoptosis, cell proliferation and neural tube closure and is often triggered by environmental factors, such as folate deficiency [193].

Folate is a central part of 1C metabolism and prevents NTDs by fuelling methylation reactions via SAM generation [186]. Choline contributes to the same methylation network through its oxidation to betaine, which remethylates homocysteine via BHMT, while folate-dependent remethylation occurs via methionine synthase, illustrating the complementary roles of these nutrients in maintaining methylation capacity [113]. Folate depletion may reduce DNA and homocysteine-mediated gene methylation, leading to hypomethylation, critical genes being unmethylated, epigenetic alternations and neural tube closure disruptions [186,190,194].

A global umbrella review of 283 records showed that maternal folic acid use was inversely related to NTD prevention at birth (RR 0.31, 95% CI 0.11–0.38) [195]. Several observational studies have also found protective relationships between higher choline intakes and reduced NTD risk [196,197,198] although some studies did not [154,199,200]. A meta-analysis of five case–control studies found that lower maternal choline intakes were associated with a higher odds ratio for NTDs (pooled estimate = 1.36; 95% CI: 1.11, 1.67) and the risk could be up to 2.36-fold in certain populations, indicating that low maternal choline status is a folate-independent risk factor for NTDs [156].

Vitamin B12 and holo-transcobalamin levels (the active vitamin B12 form) can be lower in mother–infant pairs affected by NTDs [201,202]. A systematic review of 38 studies across 14 countries globally found that vitamin B12 levels were statistically significantly lower amongst NTD cases compared with non-NTD controls (SMD = −0.23, 95% CI −0.32, −0.14), indicating that vitamin B12 supplementation is needed alongside folic acid [203]. Another review of 40 studies similarly found that vitamin B12 levels were lower and homocysteine levels were higher in mothers and infants affected by NTD [201].

Taking the evidence as a whole, alongside folate, growing evidence suggests that low maternal intakes of choline and vitamin B12 may also be associated with increased NTD risk [117]. Regarding such synergies a US case–control study including 1227 NTD cases and 7905 controls without birth defects showed greater reductions in NTD risk with concurrent higher intakes of choline and vitamin B12 in conjunction with daily periconceptional folic acid supplementation [155]. Similarly, Petersen et al. (2019) analyzed data from 164 NTD cases and 2831 controls finding that women meeting folic acid recommendations with concurrent higher intakes of vitamin B12, choline and vitamin B6 had around half the risk of a NTD-affected pregnancy [154].

### 6.2. Brain Development and Other Outcomes

**Figure 5:**
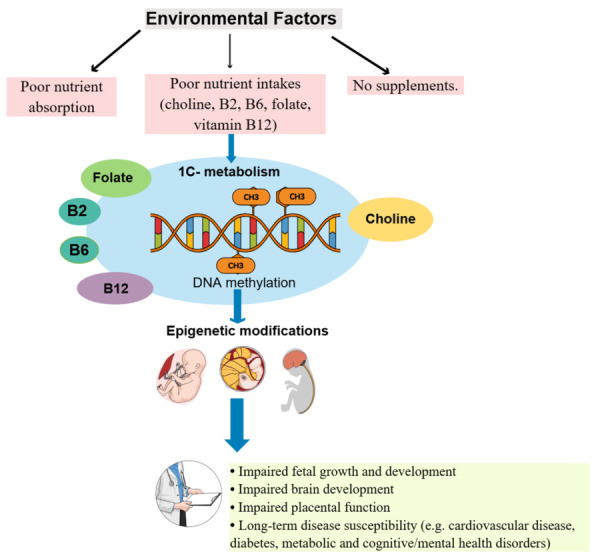
Effect of methyl-donor nutrients on neurodevelopment and future health. Source: Adapted from Socha et al. (2024) [223]. References: 1-C metabolism, methylation and development [19,20]; epigenetic modifications [8,223]; roles of nutrients [33,223,224]; fetal growth and development [8,50,120,225]; brain development [62,159,204,226]; placental function [114,227]; long-term disease susceptibility [17,228].

There is growing evidence that inadequate availability of 1C metabolism nutrients during embryonic development has an established role in NTD risk reduction, which may have lasting consequences for brain growth, development and health (Figure 5) [204,205,206]. Choline is also incorporated into phosphatidylcholine and sphingomyelin, major membrane phospholipids that collectively represent the principal storage pool of choline [207]. These phospholipids contribute to neuronal membrane formation and myelination, while phosphatidylcholine-containing lipoproteins facilitate the transport of long-chain polyunsaturated fatty acids (LC-PUFAs), including DHA and arachidonic acid, from the liver and circulation to the developing central nervous system [207,208].

Choline is increasingly recognized for its role in fetal neurogenesis and cognitive development, potentially underpinned by its involvement in acetylcholine synthesis and phosphatidylcholine-dependent cell membrane synthesis [156,163,209]. Higher intakes of choline during pregnancy have also been associated with a lower risk of adverse pregnancy outcomes (six studies; pooled OR 0.51, 95% CI 0.40–0.65), including gestational diabetes mellitus, pre-eclampsia, preterm delivery and small-for-gestational-age infants [210,211,212,213,214,215,216]. Maternal 1C metabolism nutrient status, particularly choline, also plays a critical role in placental development and fetal growth [217]. Low maternal choline status is associated with reduced placental weight, altered growth-related gene expression, and reduced placental blood flow, increasing the risk of placental hypoxia, fetal growth restriction, preterm birth, and pre-eclampsia [63,218]. In contrast, higher maternal intakes of folate and choline have been linked to lower odds of conditions such as pre-eclampsia [213].

It has been proposed that choline and vitamins B6, folate and B12 may act both independently and synergistically, with nutrition interventions before and during pregnancy potentially reducing Autism Spectrum Disorder (ASD) incidence and symptom severity [219]. Vitamin B12 deficiency may adversely affect myelin synthesis and repair, with implications for neuroprotection [89]. In addition, vitamin B12 is involved in neurotransmitter metabolism, including serotonin and dopamine synthesis, which influences mood, tcognition, and behaviour, whilst maternal vitamin B12 insufficiency has been associated with altered fetal growth, neurodevelopmental trajectories and a higher risk of offspring ASD [220,221]. Vitamin B1 is involved in myelin and neurotransmitter synthesis and forms a structural component of mitochondrial and synaptosomal membranes, with shortfalls potentially impacting fetal development [153]. Similarly, suboptimal intakes of folate and/or vitamin B2, B6i and B12 can also lead to disruptions to 1C metabolism and potentially affect brain development during early and later life [151].

Taken together, evidence indicates that the first thousand days of life represent a critical developmental window in which environmental exposures, including nutrition, can affect outcomes such as placental development, fetal organ growth, and the capacity to withstand postnatal stressors [222].

## 7. Supplementation and Fortification

It has been identified in the present publication that daily habitual intakes of folate and choline are largely inadequate amongst women of childbearing age, in Europe and beyond [77,80,229]. Intakes of vitamins B1 and B6 are often below recommended levels, indicating a potential risk of inadequacy, whereas vitamin B2 intake appears to be more adequate, although additional data are needed to confirm this [81,230]. A 2018 study evaluating daily supplementation with 400 or 800 µg folate in German women of childbearing age, conducted in Esslingen am Neckar, Germany, showed that 88% of women had insufficient RBC-folate levels to prevent birth defects, while 6.1% were classified as deficient [231]. Whilst vitamin B12 appears to be somewhat less of a concern, intakes may be lower in certain subpopulations, such as those consuming diets low in animal products [27,29].

New Zealand and Australia have now joined around 70 other countries globally by undertaking mandatory fortification of flour with folic acid for NTD prevention [232]. Canadian scientists advise that women should consider taking 0.4 mg folic acid 3 months prior to conception up until the completion of breastfeeding, with measurements of serum maternal folate levels to be taken 4–6 weeks after the commencement of supplementation amongst complex risk groups, e.g., those with genetic co-morbidities [233].

With respect to the UK, mandatory folic acid fortification of non-wholemeal wheat flour has been legislated and should come into force by late 2026—it is estimated that NTD rates should be reduced by 20% [234]. For women of reproductive age, a cut-off guide of red blood cell level threshold below 906 nmol/L indicates folate insufficiency and suboptimal protection against preventing NTDs [235]. Findings from the latest UK National Diet and Nutrition Survey show that amongst women of reproductive age (aged 16 to 49 years), 83% had red blood cell folate levels below 748 nmol per litre, indicating a heightened risk of NTDs [85].

In other countries such as Canada, it has been suggested that alongside supplementation programmes fortifying foods with vitamin B12, using strategies similar to folic acid fortification programmes could further reduce NTDs beyond the effects of folic acid alone [236]. Mandatory widespread fortification of staple foods like flour with folic acid is not implemented in many European countries, with variation in national fortification strategies and fragmented regulatory frameworks contributing to inconsistent implementation [237]. Voluntary fortification of foods (e.g., breakfast cereals with B vitamins and iron) is allowed but very uncommon for choline [237,238].

Indeed, given its limited endogenous synthesis, choline is increasingly regarded as an essential nutrient for fetal and infant development, necessitating adequate intake from diet or supplementation [239]. In Norway, an adequate intake of 400 mg/day is set for females [240,241], as also advised by the European Food Safety Authority, with 480 mg/day for pregnancy and 520 mg/day for lactation [72]. However, some scientists have questionned incremental intakes, suggesting that more than an extra 25 mg/d is needed (as per U.S. guidelines) as choline is used as a methyl donor in maternal and fetal compartments [242,243].

Choline intakes have been reported to range from 233 mg/day to 383 mg/day, generally indicating insufficient intakes during pregnancy and lactation and a need for supplementation recommendations which presently do not exist [77]. Evaluation of 180 prenatal commercial supplements further demonstrated marked heterogeneity in nutrient content, with numerous formulations lacking key essential vitamins and often supplying nutrients at levels below established recommendations [244]. Research has found supplements to be a crucial source of 1C metabolism nutrients during pregnancy and without use many expectant mothers would not meet recommended intake thresholds [245].

## 8. Public Health Implications and Future Research

### 8.1. Policy Integration

Methyl-group donors, also referred to as ‘lipotropes’, are derived from a balanced and varied diet [246]. However, a growing body of evidence emphasizes that dietary shortfalls are often apparent [28,77,80,117,155,247]. As demonstrated in the present publication, intakes of fundamental 1C metabolism nutrients are frequently suboptimal during pregnancy and do not meet the biological requirements, leading to maternal depletion of stores. As explained by Dusa et al. (2025) “*Our descendants are partly shaped by what we ate and how we lived before conception and during pregnancy*”, indicating that nutrient intakes can disrupt normal physiology [97]. Despite widespread recognition of the importance of a healthy, balanced diet, many women in European countries do not meet the recommended intakes for 1C nutrients, particularly folate, choline, and vitamin B12 [77,85,248].

Given the expanding body of evidence demonstrating associations between varying levels of dietary methyl donors and fetal growth and development and offspring health, particularly with respect to brain and neurodevelopmental outcomes and cardiometabolic health later in life, these findings carry important implications for public health policy and maternal nutrition guidance [8,163,205,220,249]. This includes:

▪ Sustained education amongst women of childbearing age about food sources of 1C metabolism nutrients to help improve habitual intakes and implications of insufficiency [250]. For more than two decades there have been public health campaigns advising periconceptional daily folic acid supplementation to reduce NTD risk, yet despite this no declines in NTDs have been reported [251]. Although diet quality improves modestly during pregnancy, overall scores remain low, highlighting the need to continue promoting healthier dietary behaviours among women of reproductive age [252].
▪ Protecting certain vulnerable population groups that may be at risk of disrupted 1C nutrient intakes. Individuals following restrictive, plant-based or vegan diets may require support, education and/or supplementation strategies to help obtain suitable choline and B vitamin intakes [28,29,253,254]. Equally, food deprivation which can be attributed to political instability, climate, food costs, lack of education and inadequate storage conditions has also been associated with micronutrient deficiencies [32].
▪ Fundamentally, it is now increasingly recognized that concurrent intakes of 1C metabolism nutrients such as choline, B2, B6, folate and vitamin B12 help regulate normal homocysteine metabolism, reducing risks of adverse pregnancy outcomes affecting mother and child [117,118,154,155,156,255] whilst helping to improve fetal neurodevelopment and growth outcomes [6,156,204,249,256]. Food fortification strategies should continue for folic acid to reduce NTD risk, with scope to consider adding other 1C metabolism nutrients such as choline and B vitamins in the future [234,236].
▪ Future preventive and therapeutic strategies may benefit from considering choline within the context of interacting nutrient networks, rather than as an isolated intervention. On some level compiling and updating public health recommendations to include folate, choline and vitamin B12 recommendations is important, although reliability and reproducibility of datasets informing such recommendations should be carefully considered [257]. This is particularly relevant given the complementary roles of these nutrients in 1C metabolism and epigenetic regulation, as well as their potential implications for lifelong health and wellbeing [38,258].
▪ Prenatal supplements can complement a healthy and balanced diet, but they should contain appropriate doses of these nutrients. Importantly, the biological effects of choline may vary according to its carrier form. Human studies have demonstrated that different choline supplements exhibit distinct absorption kinetics and metabolic profiles, including differences in trimethylamine-N-oxide (TMAO) production, with choline bitartrate producing substantially greater TMAO responses than phosphatidylcholine (amongst males) [259]. The choline Tolerable Upper Intake level for adults aged 19 years and over, including pregnant and lactating women, is 3.5 g daily [243]. Furthermore, direct comparisons of supplemental choline forms during pregnancy, including their effects on TMAO production, remain scarce.
▪ When considering the potential adverse effects of 1C supplementation, excessive folic acid intake may lead to the accumulation of unmetabolized folic acid, which has been associated with potential deleterious effects, including an increased risk of carcinogenesis [260]. However, other studies have reported that higher folate concentrations are associated with a reduced risk of adverse health outcomes, including cancer, cardiovascular disease and NTDs [261]. Although some evidence suggests that excessive folic acid intake may promote the progression of existing cancers, the scientific consensus remains nuanced. Current research points to a “dual-modulator” effect, whereby adequate intake may help prevent cancers, whereas higher doses, i.e., >1 mg/day could accelerate the growth of pre-existing lesions [262,263].
▪ One important way forward, is to promote a balanced nutrient intake whilst avoiding the perception that “more is better” [264]. EFSA concluded that there is insufficient evidence to establish a causal relationship between the dietary folate intake and cancer risk [265]. Consequently, EFSA retained the Tolerable Upper Intake (UL) for supplemental folate of 1000 μg/day, as previously established by the Scientific Committee on Food, for adults, including pregnant and lactating women [265]. Moreover, it is highly unlikely that the UL for supplemental folate is exceeded in European populations as food supplements containing high doses of folate (≥1 mg/d) are very uncommon [265].

### 8.2. Future Research

Metabolic interrelationships between choline and key B vitamins exist, so subsequently in the future it is important to examine these nutrients concurrently [117]. Beyond these, 1C metabolism nutrient interactions with nutrients such as DHA warrant further investigation [165].

Needless to say, some studies evaluating habitual dietary intakes can be subject to bias and misclassification, particularly for nutrients such as choline, which are poorly represented in dietary surveys and food composition databases [266].

It would be useful for future studies to combine dietary assessments with validated biological biomarkers of 1C metabolism nutrient status to improve accuracy, such as choline metabolites betaine, dimethylglycine, and sarcosine [242]. Many studies also rely on single-time-point dietary assessments, which may not capture variation in 1C metabolism nutrient intake across critical windows during the early life course, e.g., prenatal development, when epigenetic programming is particularly sensitive.

Clearly, variability in supplement formulations, doses, and bioavailability can also obscure interpretation and comparison across studies. Finally, polymorphisms in genes such as MTHFR, BHMT, and PEMT can modify nutrient requirements and metabolic responses, yet are not always fully accounted for [74,267,268]. To conclude, standardized DNA methylation techniques are also needed to aid comparisons across studies and reduce heterogeneity [269].

## 9. Conclusions

Science has evolved beyond a sole focus on folic acid supplementation before and during early pregnancy [146]. Taking this point into consideration, nutrients including choline and related B vitamins (B2, B6, B9 (folate) and B12) play integral roles in 1C metabolism, contributing to SAM synthesis—the principal methyl donor for epigenetic processes such as DNA methylation and thereby influencing early embryonic development and later health in life [26,114,121].

It is now increasingly recognized that choline and interconnected B vitamins involved in 1C metabolism reinforce fetal development through coordinated regulation of methyl-group supply, DNA synthesis and epigenetic programming [38,96]. Inadequate intakes may disrupt these processes, potentially increasing susceptibility to non-communicable diseases later in life, including neurocognitive disorders, cardiovascular disease, type 2 diabetes and obesity [20,204,205,270].

Overall, given the collective roles of choline and B vitamins in 1C metabolism and epigenetic regulation, this highlights the need for: (1) greater public awareness, (2) comprehensive prenatal nutrition guidelines, and (3) greater consideration of dietary sources, adequate supplementation, and potential fortification policies. Given this, promoting an adequate intake of “choline and its companions” across the early life course should be viewed as a public health priority.

## Figures and Tables

**Table 1:** Roles of choline and key B vitamins in 1C metabolism and consequences of inadequacy.

Vitamin	Roles	Consequences of Inadequacy	References
Choline	Methyl donor, DNA methylation, acetylcholine synthesis, myelination, fetal brain development, homocysteine metabolism.	Impaired DNA methylation, neurodevelopment, myelination, homocysteine metabolism and increased NTD risk.	[40,41,42]
B1 (thiamine)	Cofactor for enzymes involved in glucose metabolism & R5P generation via the pentose phosphate pathway. Nucleotide, DNA, & RNA synthesis.	Impaired energy metabolism, R5P production, and nucleotide synthesis.	[43,44,45,46]
B2 (riboflavin)	Cofactor for folate activation and homocysteine remethylation, DNA methylation & nucleotide synthesis.	Impaired folate activation, DNA methylation, and homocysteine regulation.	[26,47]
B6 (pyridoxine)	Cofactor in 1C metabolism and homocysteine regulation, DNA synthesis, methylation, neurotransmitter synthesis, myelination, nervous system development.	Impaired DNA synthesis, methylation, neurotransmitter synthesis, and nervous system development.	[38,48,49,50]
B9 (folate)	Primary donor of 1C units for nucleotide synthesis, DNA methylation, epigenetic regulation, cell proliferation, homocysteine metabolism & NTD development.	Impaired nucleotide synthesis and DNA methylation; elevated homocysteine and increased NTD risk.	[47,51,52]
B12 (cobalamin)	Cofactor in methionine synthesis & homocysteine remethylation, DNA methylation, gene regulation, myelination, nervous system development.	Impaired methylation, homocysteine remethylation, myelination, and nervous system development.	[47,50]

Key: 1C, 1 carbon; DNA, deoxyribonucleic acid; NTD, neural tube defect; R5P, ribose-5-phosphate; RNA, ribonucleic acid.

## Data Availability

No new data were created or analyzed in this study. Data sharing is not applicable to this article.

## Abbreviations

The following abbreviations are used in this manuscript:

AI	Adequate intake
AR	Average requirement
ASD	Autism Spectrum Disorder
BHMT	Betaine homocysteine methyltransferase pathway
1C	One-carbon
CH3	Methyl group
CI	Confidence interval
CVD	Cardiovascular disease
DHA	Docosahexaenoic acid
DMG	Dimethylglycine
DNA	Deoxyribonucleic acid
DOHaD	Developmental Origins of Health and Disease
EFSA	European Food Safety Authority
EPA	Eicosapentaenoic acid
GDM	Gestational diabetes mellitus
LC-PUFAs	Long-chain polyunsaturated fatty acids
MTHFR	Methyl tetrahydrofolate reductase
MTR	Gene-encoding methionine synthase
MS	Methionine synthase
5-MTHF	5-methyltetrahydrofolate
NCD	Non-communicable disease
NTD	Neural tube defect
OR	Odds Ratio
PEMT	Phosphatidylethanolamine N-methyltransferase
PRI	Population reference intake
RDA	Recommended Dietary Allowances
RNA	Ribonucleic acid
RR	Relative risk
SAH	S-adenosylhomocysteine
SAM	S-adenosylmethionine
SMD	Standard Mean Difference
THF	Tetrahydrofolate
TMAO	Trimethylamine N-oxide
UL	Tolerable Upper Intake Level

## References

[1] Li Y. (2018). Epigenetic Mechanisms Link Maternal Diets and Gut Microbiome to Obesity in the Offspring. Front. Genet..

[2] Alum E.U., Aloh H.E., Obasi D.C., Okoroh P.N., Aniokete U.C., Emeruwa A.P. (2025). Maternal Nutrition, Toxicants, and Epigenetic Programming of Obesity Across Generations. Diabetes Metab. Syndr. Obes..

[3] Stephenson J., Heslehurst N., Hall J., Schoenaker D., Hutchinson J., Cade J.E., Poston L., Barrett G., Crozier S.R., Barker M. (2018). Before the beginning: Nutrition and lifestyle in the preconception period and its importance for future health. Lancet.

[4] Jain S., Maheshwari A., Jain S.K. (2022). Maternal Nutrition and Fetal/Infant Development. Clin. Perinatol..

[5] Talebi S., Kianifar H.R., Mehdizadeh A. (2024). Nutritional requirements in pregnancy and lactation. Clin. Nutr. ESPEN.

[6] Na X., Mackean P.P., Cape G.A., Johnson J.W., Ou X. (2024). Maternal Nutrition during Pregnancy and Offspring Brain Development: Insights from Neuroimaging. Nutrients.

[7] Randunu R.S., Bertolo R.F. (2020). The Effects of Maternal and Postnatal Dietary Methyl Nutrients on Epigenetic Changes that Lead to Non-Communicable Diseases in Adulthood. Int. J. Mol. Sci..

[8] McGee M., Bainbridge S., Fontaine-Bisson B. (2018). A crucial role for maternal dietary methyl donor intake in epigenetic programming and fetal growth outcomes. Nutr. Rev..

[9] Zhou Y., Xu Y. (2023). Nutrition and Metabolism in the First 1000 Days of Life. Nutrients.

[10] Beluska-Turkan K., Korczak R., Hartell B., Moskal K., Maukonen J., Alexander D.E., Salem N., Harkness L., Ayad W., Szaro J. (2019). Nutritional Gaps and Supplementation in the First 1000 Days. Nutrients.

[11] Zuccarello D., Sorrentino U., Brasson V., Marin L., Piccolo C., Capalbo A., Andrisani A., Cassina M. (2022). Epigenetics of pregnancy: Looking beyond the DNA code. J. Assist. Reprod. Genet..

[12] de Boo H.A., Harding J.E. (2006). The developmental origins of adult disease (Barker) hypothesis. Aust. N. Z. J. Obstet. Gynaecol..

[13] Buklijas T., Al-Gailani S. (2023). A fetus in the world: Physiology, epidemiology, and the making of fetal origins of adult disease. Hist. Philos. Life Sci..

[14] Cai S., Quan S., Yang G., Ye Q., Chen M., Yu H., Wang G., Wang Y., Zeng X., Qiao S. (2021). One Carbon Metabolism and Mammalian Pregnancy Outcomes. Mol. Nutr. Food Res..

[15] Simeoni U., Armengaud J.B., Siddeek B., Tolsa J.F. (2018). Perinatal Origins of Adult Disease. Neonatology.

[16] Reynolds L.P., Diniz W.J.S., Crouse M.S., Caton J.S., Dahlen C.R., Borowicz P.P., Ward A.K. (2022). Maternal nutrition and developmental programming of offspring. Reprod. Fertil. Dev..

[17] Hales C.N., Barker D.J. (2001). The thrifty phenotype hypothesis. Br. Med. Bull..

[18] Barker D.J. (2004). The developmental origins of adult disease. J. Am. Coll. Nutr..

[19] Korsmo H.W., Jiang X. (2021). One carbon metabolism and early development: A diet-dependent destiny. Trends Endocrinol. Metab..

[20] Bokor S., Csolle I., Felso R., Vass R.A., Funke S., Ertl T., Molnar D. (2024). Dietary nutrients during gestation cause obesity and related metabolic changes by altering DNA methylation in the offspring. Front. Endocrinol..

[21] Rees W.D. (2019). Interactions between nutrients in the maternal diet and the implications for the long-term health of the offspring. Proc. Nutr. Soc..

[22] Agrawal P., Kaur J., Singh J., Rasane P., Sharma K., Bhadariya V., Kaur S., Kumar V. (2024). Genetics, Nutrition, and Health: A New Frontier in Disease Prevention. J. Am. Nutr. Assoc..

[23] Wu M.M., Yang F. (2017). Research advances in the association between maternal intake of methyl donor nutrients during pregnancy and DNA methylation in offspring. Zhongguo Dang Dai Er Ke Za Zhi.

[24] Lacagnina S. (2020). The Developmental Origins of Health and Disease (DOHaD). Am. J. Lifestyle Med..

[25] Godfrey K.M., Lillycrop K.A., Burdge G.C., Gluckman P.D., Hanson M.A. (2007). Epigenetic mechanisms and the mismatch concept of the developmental origins of health and disease. Pediatr. Res..

[26] Naninck E.F.G., Stijger P.C., Brouwer-Brolsma E.M. (2019). The Importance of Maternal Folate Status for Brain Development and Function of Offspring. Adv. Nutr..

[27] Fulgoni V.L., Agarwal S., Marinangeli C.P.F., Miller K. (2024). Impact of Plant Protein Intakes on Nutrient Adequacy in the US. Nutrients.

[28] Neufingerl N., Eilander A. (2021). Nutrient Intake and Status in Adults Consuming Plant-Based Diets Compared to Meat-Eaters: A Systematic Review. Nutrients.

[29] Obeid R., Heil S.G., Verhoeven M.M.A., van den Heuvel E., de Groot L., Eussen S. (2019). Vitamin B12 Intake From Animal Foods, Biomarkers, and Health Aspects. Front. Nutr..

[30] Zuk E., Nikrandt G., Chmurzynska A. (2024). Dietary choline intake in European and non-european populations: Current status and future trends-a narrative review. Nutr. J..

[31] Paulino D.S.M., Pinho-Pompeu M., Assumpcao D., Kasawara K.T., Surita F.G. (2022). Dietary intake profile in high-risk pregnant women according to the degree of food processing. J. Matern. Fetal Neonatal Med..

[32] Lopes S.O., Abrantes L.C.S., Azevedo F.M., Morais N.S., Morais D.C., Goncalves V.S.S., Fontes E.A.F., Franceschini S., Priore S.E. (2023). Food Insecurity and Micronutrient Deficiency in Adults: A Systematic Review and Meta-Analysis. Nutrients.

[33] Cochrane K.M., Williams B.A., Elango R., Barr S.I., Karakochuk C.D. (2022). Pregnancy-induced alterations of 1-carbon metabolism and significance for maternal nutrition requirements. Nutr. Rev..

[34] Yajnik C.S., Deshmukh U.S. (2012). Fetal programming: Maternal nutrition and role of one-carbon metabolism. Rev. Endocr. Metab. Disord..

[35] Sukhera J. (2022). Narrative Reviews: Flexible, Rigorous, and Practical. J. Grad. Med. Educ..

[36] van Mil N.H., Bouwland-Both M.I., Stolk L., Verbiest M.M., Hofman A., Jaddoe V.W., Verhulst F.C., Eilers P.H., Uitterlinden A.G., Steegers E.A. (2014). Determinants of maternal pregnancy one-carbon metabolism and newborn human DNA methylation profiles. Reproduction.

[37] Petrova B., Maynard A.G., Wang P., Kanarek N. (2023). Regulatory mechanisms of one-carbon metabolism enzymes. J. Biol. Chem..

[38] Friso S., Udali S., De Santis D., Choi S.W. (2017). One-carbon metabolism and epigenetics. Mol. Asp. Med..

[39] Kareem O., Nisar S., Tanvir M., Muzaffer U., Bader G.N. (2023). Thiamine deficiency in pregnancy and lactation: Implications and present perspectives. Front. Nutr..

[40] Blusztajn J.K., Mellott T.J. (2012). Choline nutrition programs brain development via DNA and histone methylation. Cent. Nerv. Syst. Agents Med. Chem..

[41] Blusztajn J.K., Slack B.E., Mellott T.J. (2017). Neuroprotective Actions of Dietary Choline. Nutrients.

[42] Bekdash R.A. (2019). Neuroprotective Effects of Choline and Other Methyl Donors. Nutrients.

[43] Cornell University Thiamine Biochemsitry. http://thiamine.dnr.cornell.edu/Thiamine_biochemistry.html.

[44] TeSlaa T., Ralser M., Fan J., Rabinowitz J.D. (2023). The pentose phosphate pathway in health and disease. Nat. Metab..

[45] Guilland J.C. (2013). Vitamin B1 (thiamine). Rev. Prat..

[46] Kazmierczak-Baranska J., Halczuk K., Karwowski B.T. (2025). Thiamine (Vitamin B1)-An Essential Health Regulator. Nutrients.

[47] Lyon P., Strippoli V., Fang B., Cimmino L. (2020). B Vitamins and One-Carbon Metabolism: Implications in Human Health and Disease. Nutrients.

[48] Selhub J. (2002). Folate, vitamin B12 and vitamin B6 and one carbon metabolism. J. Nutr. Health Aging.

[49] Kennedy D.O. (2016). B Vitamins and the Brain: Mechanisms, Dose and Efficacy—A Review. Nutrients.

[50] Kalhan S.C. (2016). One carbon metabolism in pregnancy: Impact on maternal, fetal and neonatal health. Mol. Cell. Endocrinol..

[51] Ducker G.S., Rabinowitz J.D. (2017). One-Carbon Metabolism in Health and Disease. Cell Metab..

[52] Pentieva K., Caffrey A., Duffy B., Ward M., Clements M., Kerr M., McNulty H. (2025). B-vitamins and one-carbon metabolism during pregnancy: Health impacts and challenges. Proc. Nutr. Soc..

[53] Padmanabhan N., Watson E.D. (2013). Lessons from the one-carbon metabolism: Passing it along to the next generation. Reprod. BioMed Online.

[54] Reynolds E.H. (2025). Folate, vitamin B12, one carbon metabolism and the nervous system: Excess folic acid is potentially harmful. J. Neurol. Sci..

[55] Alvarez-Mejia D., Rodas J.A., Leon-Rojas J.E. (2025). From Womb to Mind: Prenatal Epigenetic Influences on Mental Health Disorders. Int. J. Mol. Sci..

[56] Clare C.E., Brassington A.H., Kwong W.Y., Sinclair K.D. (2019). One-Carbon Metabolism: Linking Nutritional Biochemistry to Epigenetic Programming of Long-Term Development. Annu. Rev. Anim. Biosci..

[57] Ali M.A., Hafez H.A., Kamel M.A., Ghamry H.I., Shukry M., Farag M.A. (2022). Dietary Vitamin B Complex: Orchestration in Human Nutrition throughout Life with Sex Differences. Nutrients.

[58] Dai C., Fei Y., Li J., Shi Y., Yang X. (2021). A Novel Review of Homocysteine and Pregnancy Complications. BioMed Res. Int..

[59] Bala R., Verma R., Verma P., Singh V., Yadav N., Rajender S., Agrawal N.R., Singh K. (2021). Hyperhomocysteinemia and low vitamin B12 are associated with the risk of early pregnancy loss: A clinical study and meta-analyses. Nutr. Res..

[60] Sanders L.M., Zeisel S.H. (2007). Choline: Dietary Requirements and Role in Brain Development. Nutr. Today.

[61] Bala R., Verma R., Budhwar S., Prakash N., Sachan S. (2022). Fetal hyperhomocysteinemia is associated with placental inflammation and early breakdown of maternal-fetal tolerance in pre-term birth. Am. J. Reprod. Immunol..

[62] Derbyshire E., Obeid R. (2020). Choline, Neurological Development and Brain Function: A Systematic Review Focusing on the First 1000 Days. Nutrients.

[63] Gutherz O.R., Li Q., Deyssenroth M., Wainwright H., Jacobson J.L., Meintjes E.M., Chen J., Jacobson S.W., Carter R.C. (2025). The roles of maternal one-carbon metabolism and placental imprinted gene expression in placental development and somatic growth in a longitudinal birth cohort. Placenta.

[64] EFSA Dietary Reference Values. https://www.efsa.europa.eu/en/topics/topic/dietary-reference-values.

[65] Jouanne M., Oddoux S., Noel A., Voisin-Chiret A.S. (2021). Nutrient Requirements during Pregnancy and Lactation. Nutrients.

[66] Drews K. (2015). Folate metabolism—Epigenetic role of choline and vitamin B12 during pregnancy. Ginekol. Pol..

[67] EFSA (2016). Dietary Reference Values for vitamin B6. EFSA J..

[68] EFSA (2016). Dietary reference values for thiamin. EFSA J..

[69] EFSA (2017). Dietary Reference Values for riboflavin. EFSA J..

[70] EFSA (2014). Scientific Opinion on Dietary Reference Values for folate. EFSA Panel on Dietetic Products, Nutrition and Allergies (NDA). EFSA J..

[71] EFSA (2015). Scientific Opinion on Dietary Reference Values for cobalamin (vitamin B12). EFSA J..

[72] EFSA (2016). Dietary Reference Values for choline. EFSA Panel on Dietetic Products, Nutrition and Allergies (NDA). EFSA J..

[73] Zhu X., Song J., Mar M.H., Edwards L.J., Zeisel S.H. (2003). Phosphatidylethanolamine N-methyltransferase (PEMT) knockout mice have hepatic steatosis and abnormal hepatic choline metabolite concentrations despite ingesting a recommended dietary intake of choline. Biochem. J..

[74] Zeisel S.H. (2007). Gene response elements, genetic polymorphisms and epigenetics influence the human dietary requirement for choline. IUBMB Life.

[75] da Costa K.A., Kozyreva O.G., Song J., Galanko J.A., Fischer L.M., Zeisel S.H. (2006). Common genetic polymorphisms affect the human requirement for the nutrient choline. FASEB J..

[76] Saito S., Iida A., Sekine A., Miura Y., Sakamoto T., Ogawa C., Kawauchi S., Higuchi S., Nakamura Y. (2001). Identification of 197 genetic variations in six human methyltranferase genes in the Japanese population. J. Hum. Genet..

[77] Derbyshire E., Obeid R., Schon C. (2021). Habitual Choline Intakes across the Childbearing Years: A Review. Nutrients.

[78] Probst Y., Guan V., Neale E. (2019). Development of a Choline Database to Estimate Australian Population Intakes. Nutrients.

[79] Wu B.T., Dyer R.A., King D.J., Richardson K.J., Innis S.M. (2012). Early second trimester maternal plasma choline and betaine are related to measures of early cognitive development in term infants. PLoS ONE.

[80] Redruello Requejo M., Carretero Krug A., Samaniego Vaesken M.L., Partearroyo Cediel T., Varela Moreiras G. (2021). Quantification, dietary intake adequacy, and food sources of nutrients involved in the methionine-methylation cycle (choline, betaine, folate, vitamin B6 and vitamin B12) in pregnant women in Spain. Nutr. Hosp..

[81] Aparicio E., Jardi C., Bedmar C., Palleja M., Basora J., Arija V., The ECLIPSES Study Group (2020). Nutrient Intake during Pregnancy and Post-Partum: ECLIPSES Study. Nutrients.

[82] Jarvie E.M., Lovegrove J.A., Weech M., Freeman D.J., Meyer B.J. (2025). Dietary Micronutrient Intake During Pregnancy Is Suboptimal in a Group of Healthy Scottish Women, Irrespective of Maternal Body Mass Index. Nutrients.

[83] Devalia V., Hamilton M.S., Molloy A.M., British Committee for Standards in Haematology (2014). Guidelines for the diagnosis and treatment of cobalamin and folate disorders. Br. J. Haematol..

[84] Sukumar N., Adaikalakoteswari A., Venkataraman H., Maheswaran H., Saravanan P. (2016). Vitamin B12 status in women of childbearing age in the UK and its relationship with national nutrient intake guidelines: Results from two National Diet and Nutrition Surveys. BMJ Open.

[85] OHID (2025). National Diet and Nutrition Survey: The National Diet and Nutrition Survey Assesses the Diet, Nutrient Intake and Nutritional Status of the General Population of the UK.

[86] Rooney D.J., Conway M., O’Keeffe L.M., McDonnell C.M., Bartels H.C., Yelverton C., Segurado R., Mehegan J., McAuliffe F.M. (2024). Dietary intakes of iron, folate, and vitamin B12 during pregnancy and correlation with maternal hemoglobin and fetal growth: Findings from the ROLO longitudinal birth cohort study. Arch. Gynecol. Obstet..

[87] Shipton M.J., Thachil J. (2015). Vitamin B12 deficiency—A 21st century perspective. Clin. Med..

[88] Green R., Datta Mitra A. (2017). Megaloblastic Anemias: Nutritional and Other Causes. Med. Clin. N. Am..

[89] Green R., Miller J.W. (2022). Vitamin B12 deficiency. Vitam. Horm..

[90] Zeisel S.H., Da Costa K.A., Franklin P.D., Alexander E.A., Lamont J.T., Sheard N.F., Beiser A. (1991). Choline, an essential nutrient for humans. FASEB J..

[91] Moore L.D., Le T., Fan G. (2013). DNA methylation and its basic function. Neuropsychopharmacology.

[92] Das J., Maitra A. (2021). Maternal DNA Methylation During Pregnancy: A Review. Reprod. Sci..

[93] Jain R., Epstein J.A. (2024). Epigenetics. Adv. Exp. Med. Biol..

[94] Felix J.F., Cecil C.A.M. (2019). Population DNA methylation studies in the Developmental Origins of Health and Disease (DOHaD) framework. J. Dev. Orig. Health Dis..

[95] Zheng J., Xiao X., Zhang Q., Yu M. (2014). DNA methylation: The pivotal interaction between early-life nutrition and glucose metabolism in later life. Br. J. Nutr..

[96] Dominguez-Salas P., Cox S.E., Prentice A.M., Hennig B.J., Moore S.E. (2012). Maternal nutritional status, C_1_ metabolism and offspring DNA methylation: A review of current evidence in human subjects. Proc. Nutr. Soc..

[97] Dusa F., Vellai T., Sipos M. (2025). Nutrition and DNA Methylation: How Dietary Methyl Donors Affect Reproduction and Aging. Dietetics.

[98] CDC (2025). Epigenetic, Health, and Disease. https://www.cdc.gov/genomics-and-health/epigenetics/index.html.

[99] Heijmans B.T., Tobi E.W., Stein A.D., Putter H., Blauw G.J., Susser E.S., Slagboom P.E., Lumey L.H. (2008). Persistent epigenetic differences associated with prenatal exposure to famine in humans. Proc. Natl. Acad. Sci. USA.

[100] Tobi E.W., Lumey L.H., Talens R.P., Kremer D., Putter H., Stein A.D., Slagboom P.E., Heijmans B.T. (2009). DNA methylation differences after exposure to prenatal famine are common and timing- and sex-specific. Hum. Mol. Genet..

[101] Tobi E.W., Slieker R.C., Luijk R., Dekkers K.F., Stein A.D., Xu K.M., Biobank-Based Integrative Omics Studies Consortium, Slagboom P.E., van Zwet E.W., Lumey L.H. (2018). DNA methylation as a mediator of the association between prenatal adversity and risk factors for metabolic disease in adulthood. Sci. Adv..

[102] Roseboom T.J. (2019). Epidemiological evidence for the developmental origins of health and disease: Effects of prenatal undernutrition in humans. J. Endocrinol..

[103] Jiang X., Yan J., West A.A., Perry C.A., Malysheva O.V., Devapatla S., Pressman E., Vermeylen F., Caudill M.A. (2012). Maternal choline intake alters the epigenetic state of fetal cortisol-regulating genes in humans. FASEB J..

[104] Li Y.M., Xiao X., Wang J., Liu Y.X., Pan X.F., Yu H.B., Luo J.Y., Luo M.Y. (2024). Genetic Variations and Nonalcoholic Fatty Liver Disease: Field Synopsis, Systematic Meta-Analysis, and Epidemiological Evidence. BioMed Environ. Sci..

[105] Tacke F., Horn P., Wong V.W., Ratziu V., Bugianesi E., Francque S., Zelber-Sagi S., Valenti L., Roden M., Schick F. (2024). EASL–EASD–EASO Clinical Practice Guidelines on the management of metabolic dysfunction-associated steatotic liver disease (MASLD). J. Hepatol..

[106] van Otterdijk S.D., Klett H., Boerries M., Michels K.B. (2023). The impact of pre-pregnancy folic acid intake on placental DNA methylation in a fortified cohort. FASEB J..

[107] Wilson A., Platt R., Wu Q., Leclerc D., Christensen B., Yang H., Gravel R.A., Rozen R. (1999). A common variant in methionine synthase reductase combined with low cobalamin (vitamin B12) increases risk for spina bifida. Mol. Genet. Metab..

[108] Lievers K.J., Kluijtmans L.A., Heil S.G., Boers G.H., Verhoef P., Den Heijer M., Trijbels F.J., Blom H.J. (2003). Cystathionine beta-synthase polymorphisms and hyperhomocysteinaemia: An association study. Eur. J. Hum. Genet..

[109] Jhee K.H., Kruger W.D. (2005). The role of cystathionine beta-synthase in homocysteine metabolism. Antioxid. Redox Signal..

[110] Stover P.J. (2011). Polymorphisms in 1-carbon metabolism, epigenetics and folate-related pathologies. J. Nutr. Nutr..

[111] Zeisel S.H. (2011). The supply of choline is important for fetal progenitor cells. Semin. Cell Dev. Biol..

[112] Ghazi T., Arumugam T., Foolchand A., Chuturgoon A.A. (2020). The Impact of Natural Dietary Compounds and Food-Borne Mycotoxins on DNA Methylation and Cancer. Cells.

[113] Bortz J., Obeid R. (2025). The Shuttling of Methyl Groups Between Folate and Choline Pathways. Nutrients.

[114] Steane S.E., Cuffe J.S.M., Moritz K.M. (2023). The role of maternal choline, folate and one-carbon metabolism in mediating the impact of prenatal alcohol exposure on placental and fetal development. J. Physiol..

[115] Zeisel S.H. (2009). Importance of methyl donors during reproduction. Am. J. Clin. Nutr..

[116] Jankovic-Karasoulos T., Furness D.L., Leemaqz S.Y., Dekker G.A., Grzeskowiak L.E., Grieger J.A., Andraweera P.H., McCullough D., McAninch D., McCowan L.M. (2021). Maternal folate, one-carbon metabolism and pregnancy outcomes. Matern. Child Nutr..

[117] Obeid R., Holzgreve W., Pietrzik K. (2022). Folate, Choline, and Vitamin B12 Supplementation for Pre-Conceptional and Pregnant Women. Ther. Umsch..

[118] Li K., Wahlqvist M.L., Li D. (2016). Nutrition, One-Carbon Metabolism and Neural Tube Defects: A Review. Nutrients.

[119] Obeid R. (2013). The metabolic burden of methyl donor deficiency with focus on the betaine homocysteine methyltransferase pathway. Nutrients.

[120] Zeisel S. (2017). Choline, Other Methyl-Donors and Epigenetics. Nutrients.

[121] Froese D.S., Fowler B., Baumgartner M.R. (2019). Vitamin B_12_, folate, and the methionine remethylation cycle-biochemistry, pathways, and regulation. J. Inherit. Metab. Dis..

[122] Lever M., McEntyre C.J., George P.M., Chambers S.T. (2017). Is N,N-dimethylglycine N-oxide a choline and betaine metabolite?. Biol. Chem..

[123] Castillo L.F., Pelletier C.M., Heyden K.E., Field M.S. (2025). New Insights into Folate-Vitamin B_12_ Interactions. Annu. Rev. Nutr..

[124] Vidmar Golja M., Smid A., Karas Kuzelicki N., Trontelj J., Gersak K., Mlinaric-Rascan I. (2020). Folate Insufficiency Due to MTHFR Deficiency Is Bypassed by 5-Methyltetrahydrofolate. J. Clin. Med..

[125] Banerjee R.V., Matthews R.G. (1990). Cobalamin-dependent methionine synthase. FASEB J..

[126] Scott J.M. (1999). Folate and vitamin B12. Proc. Nutr. Soc..

[127] Ramakrishnan S., Sulochana K.N., Lakshmi S., Selvi R., Angayarkanni N. (2006). Biochemistry of homocysteine in health and diseases. Indian J. Biochem. Biophys..

[128] Franco C.N., Seabrook L.J., Nguyen S.T., Leonard J.T., Albrecht L.V. (2022). Simplifying the B Complex: How Vitamins B6 and B9 Modulate One Carbon Metabolism in Cancer and Beyond. Metabolites.

[129] Chen V., Schwartz J.L., Cho C.E. (2023). Folate and Choline: Does It Take Two to Tango in Early Programming of Disease?. Lifestyle Genom..

[130] Nogueira-de-Almeida C.A., Zotarelli-Filho I.J., Nogueirade-Almeida M.E., Souza C.G., Kemp V.L., Ramos W.S. (2023). Neuronutrients and Central Nervous System: A Systematic Review. Cent. Nerv. Syst. Agents Med. Chem..

[131] Krishnaveni G.V., Veena S.R., Johnson M., Kumaran K., Jones A., Bhat D.S., Yajnik C.S., Fall C.H.D. (2020). Maternal B12, Folate and Homocysteine Concentrations and Offspring Cortisol and Cardiovascular Responses to Stress. J. Clin. Endocrinol. Metab..

[132] Benitez B.C. (2024). Better Early: Critical Windows in Brain and Cognitive Development. Nestle Nutr. Inst. Workshop Ser..

[133] Cai S., Quan S., Yang G., Chen M., Ye Q., Wang G., Yu H., Wang Y., Qiao S., Zeng X. (2021). Nutritional Status Impacts Epigenetic Regulation in Early Embryo Development: A Scoping Review. Adv. Nutr..

[134] Lee H.S. (2015). Impact of Maternal Diet on the Epigenome during In Utero Life and the Developmental Programming of Diseases in Childhood and Adulthood. Nutrients.

[135] Renz H., Adkins B.D., Bartfeld S., Blumberg R.S., Farber D.L., Garssen J., Ghazal P., Hackam D.J., Marsland B.J., McCoy K.D. (2018). The neonatal window of opportunity-early priming for life. J. Allergy Clin. Immunol..

[136] Hochberg Z., Feil R., Constancia M., Fraga M., Junien C., Carel J.C., Boileau P., Le Bouc Y., Deal C.L., Lillycrop K. (2011). Child health, developmental plasticity, and epigenetic programming. Endocr. Rev..

[137] NSCDC. National Scientific Council on the Developing Child (2010). Early Experiences Can Alter Gene Expression and Affect Long-Term Development: Working Paper No. 10. https://developingchild.harvard.edu/wp-content/uploads/2024/10/Early-Experiences-Can-Alter-Gene-Expression-and-Affect-Long-Term-Development.pdf.

[138] Bakulski K.M., Blostein F., London S.J. (2023). Linking Prenatal Environmental Exposures to Lifetime Health with Epigenome-Wide Association Studies: State-of-the-Science Review and Future Recommendations. Environ. Health Perspect..

[139] Symonds M.E., Stephenson T., Gardner D.S., Budge H. (2007). Long-term effects of nutritional programming of the embryo and fetus: Mechanisms and critical windows. Reprod. Fertil. Dev..

[140] Donovan M., Cascella M. Embryology, Weeks 6–8. https://www.ncbi.nlm.nih.gov/books/NBK563181/.

[141] Sadler T.W. (2000). Susceptible periods during embryogenesis of the heart and endocrine glands. Environ. Health Perspect..

[142] Derbyshire E. (2011). Diet and Pregnancy Outcome. Nutrition in the Childbearing Years.

[143] Selevan S.G., Kimmel C.A., Mendola P. (2000). Identifying critical windows of exposure for children’s health. Environ. Health Perspect..

[144] Parsons E., Rifas-Shiman S.L., Bozack A.K., Baccarelli A.A., DeMeo D.L., Hivert M.F., Godderis L., Duca R.C., Oken E., Cardenas A. (2023). Prenatal trimester-specific intake of micronutrients: Global DNA methylation and hydroxymethylation at birth and persistence in childhood. J. Dev. Orig. Health Dis..

[145] Seyoum Tola F. (2024). The concept of folic acid supplementation and its role in prevention of neural tube defect among pregnant women: PRISMA. Medicine.

[146] SACN (2017). Folic Acid: Updated SACN Recommendations.

[147] Santos C., Marshall A.R., Murray A., Metcalfe K., Narayan P., de Castro S.C.P., Maniou E., Greene N.D.E., Galea G.L., Copp A.J. (2024). Spinal neural tube formation and tail development in human embryos. eLife.

[148] Houston M. (2012). Taking folic acid at start of pregnancy seems to lower risk of cleft lip and palate. BMJ.

[149] van Vliet M.M., Schoenmakers S., Willemsen S.P., Sinclair K.D., Steegers-Theunissen R.P.M. (2025). First-trimester maternal folate and vitamin B12 concentrations and their associations with first-trimester placental growth: The Rotterdam Periconception Cohort. Hum. Reprod..

[150] Gao J., Lou Y., He W., Xu K., Zhan X., Tong J., Zhai H. (2025). Folic acid ameliorates placental structure and function in fetal growth restriction via epigenetic modifications. Clin. Epigenet..

[151] Caffrey A., Lamers Y., Murphy M.M., Letourneau N., Irwin R.E., Pentieva K., Ward M., Tan A., Rojas-Gomez A., Santos-Calderon L.A. (2023). Epigenetic effects of folate and related B vitamins on brain health throughout life: Scientific substantiation and translation of the evidence for health improvement strategies. Nutr. Bull..

[152] McGarel C., Pentieva K., Strain J.J., McNulty H. (2015). Emerging roles for folate and related B-vitamins in brain health across the lifecycle. Proc. Nutr. Soc..

[153] Kloss O., Eskin N.A.M., Suh M. (2018). Thiamin deficiency on fetal brain development with and without prenatal alcohol exposure. Biochem. Cell Biol..

[154] Petersen J.M., Parker S.E., Crider K.S., Tinker S.C., Mitchell A.A., Werler M.M. (2019). One-Carbon Cofactor Intake and Risk of Neural Tube Defects Among Women Who Meet Folic Acid Recommendations: A Multicenter Case-Control Study. Am. J. Epidemiol..

[155] Petersen J.M., Smith-Webb R.S., Shaw G.M., Carmichael S.L., Desrosiers T.A., Nestoridi E., Darling A.M., Parker S.E., Politis M.D., Yazdy M.M. (2023). Periconceptional intakes of methyl donors and other micronutrients involved in one-carbon metabolism may further reduce the risk of neural tube defects in offspring: A United States population-based case-control study of women meeting the folic acid recommendations. Am. J. Clin. Nutr..

[156] Obeid R., Derbyshire E., Schon C. (2022). Association between Maternal Choline, Fetal Brain Development, and Child Neurocognition: Systematic Review and Meta-Analysis of Human Studies. Adv. Nutr..

[157] Jiang X., West A.A., Caudill M.A. (2014). Maternal choline supplementation: A nutritional approach for improving offspring health?. Trends Endocrinol. Metab..

[158] Jiang X., Yan J., West A., Perry C., Malysheva O., Bar H. (2012). Pregnancy status and choline intake alter DNA integrity, epigenetic marks and gene expression. FASEB J..

[159] Zeisel S.H. (2006). The fetal origins of memory: The role of dietary choline in optimal brain development. J. Pediatr..

[160] King R.G., Gude N.M., Krishna B.R., Chen S., Brennecke S.P., Boura A.L., Rook T.J. (1991). Human placental acetylcholine. Reprod. Fertil. Dev..

[161] Portal I., Clerc T., Sbarra V., Portugal H., Pauli A.M., Lafont H., Tuchweber B., Yousef I., Chanussot F. (1993). Importance of high-density lipoprotein-phosphatidylcholine in secretion of phospholipid and cholesterol in bile. Am. J. Physiol..

[162] Massey J.B. (2001). Interaction of ceramides with phosphatidylcholine, sphingomyelin and sphingomyelin/cholesterol bilayers. Biochim. Biophys. Acta.

[163] Mujica-Coopman M.F., Paules E.M., Trujillo-Gonzalez I. (2024). The role of prenatal choline and its impact on neurodevelopmental disorders. Front. Nutr..

[164] Caudill M.A., Strupp B.J., Muscalu L., Nevins J.E.H., Canfield R.L. (2018). Maternal choline supplementation during the third trimester of pregnancy improves infant information processing speed: A randomized, double-blind, controlled feeding study. FASEB J..

[165] Mun J.G., Legette L.L., Ikonte C.J., Mitmesser S.H. (2019). Choline and DHA in Maternal and Infant Nutrition: Synergistic Implications in Brain and Eye Health. Nutrients.

[166] Basak S., Mallick R., Banerjee A., Pathak S., Duttaroy A.K. (2021). Maternal Supply of Both Arachidonic and Docosahexaenoic Acids Is Required for Optimal Neurodevelopment. Nutrients.

[167] Postle A.D., Al M.D., Burdge G.C., Hornstra G. (1995). The composition of individual molecular species of plasma phosphatidylcholine in human pregnancy. Early Hum. Dev..

[168] Burdge G.C., Postle A.D. (1994). Hepatic phospholipid molecular species in the guinea pig. Adaptations to pregnancy. Lipids.

[169] Bernhard W., Bockmann K., Maas C., Mathes M., Hovelmann J., Shunova A., Hund V., Schleicher E., Poets C.F., Franz A.R. (2020). Combined choline and DHA supplementation: A randomized controlled trial. Eur. J. Nutr..

[170] Mandaviya P.R., Stolk L., Heil S.G. (2014). Homocysteine and DNA methylation: A review of animal and human literature. Mol. Genet. Metab..

[171] King J.H., Kwan S.T.C., Bae S., Klatt K.C., Yan J., Malysheva O.V., Jiang X., Roberson M.S., Caudill M.A. (2019). Maternal choline supplementation alters vitamin B-12 status in human and murine pregnancy. J. Nutr. Biochem..

[172] Narváez J., Maldonado G., Intriago M., Cárdenas J., Guerrero R., Neyro J.L., Ríos C. (2020). Role of homocysteine and vitamin B in bone metabolism. Rev. Colomb. Reumatol..

[173] Friesen R.W., Novak E.M., Hasman D., Innis S.M. (2007). Relationship of dimethylglycine, choline, and betaine with oxoproline in plasma of pregnant women and their newborn infants. J. Nutr..

[174] Li M., Ren R., Wang K., Wang S., Chow A., Yang A.K., Lu Y., Leo C. (2025). Effects of B Vitamins on Homocysteine Lowering and Thrombotic Risk Reduction—A Review of Randomized Controlled Trials Published Since January 1996. Nutrients.

[175] Stead L.M., Jacobs R.L., Brosnan M.E., Brosnan J.T. (2004). Methylation demand and homocysteine metabolism. Adv. Enzym. Regul..

[176] de la Calle M., Usandizaga R., Sancha M., Magdaleno F., Herranz A., Cabrillo E. (2003). Homocysteine, folic acid and B-group vitamins in obstetrics and gynaecology. Eur. J. Obstet. Gynecol. Reprod. Biol..

[177] Zaric B.L., Obradovic M., Bajic V., Haidara M.A., Jovanovic M., Isenovic E.R. (2019). Homocysteine and Hyperhomocysteinaemia. Curr. Med. Chem..

[178] Son P., Lewis L. (2025). Hyperhomocysteinemia.

[179] Marroncini G., Martinelli S., Menchetti S., Bombardiere F., Martelli F.S. (2024). Hyperhomocysteinemia and Disease-Is 10 mumol/L a Suitable New Threshold Limit?. Int. J. Mol. Sci..

[180] Steegers-Theunissen R.P., Obermann-Borst S.A., Kremer D., Lindemans J., Siebel C., Steegers E.A., Slagboom P.E., Heijmans B.T. (2009). Periconceptional maternal folic acid use of 400 microg per day is related to increased methylation of the IGF2 gene in the very young child. PLoS ONE.

[181] Bergen N.E., Jaddoe V.W., Timmermans S., Hofman A., Lindemans J., Russcher H., Raat H., Steegers-Theunissen R.P., Steegers E.A. (2012). Homocysteine and folate concentrations in early pregnancy and the risk of adverse pregnancy outcomes: The Generation R Study. BJOG.

[182] Steegers-Theunissen R.P., Boers G.H., Trijbels F.J., Finkelstein J.D., Blom H.J., Thomas C.M., Borm G.F., Wouters M.G., Eskes T.K. (1994). Maternal hyperhomocysteinemia: A risk factor for neural-tube defects?. Metabolism.

[183] Steegers-Theunissen R.P., Van Iersel C.A., Peer P.G., Nelen W.L., Steegers E.A. (2004). Hyperhomocysteinemia, pregnancy complications, and the timing of investigation. Obstet. Gynecol..

[184] Li S., Wu Y., Gao Y., Tian A., Yao M., Meng F., Liang F., Li Y., Zhang C., Luo X. (2026). Maternal hyperhomocysteinemia induces fetal growth restriction by suppressing angiogenesis at the maternal-fetal interface. Cell Biosci..

[185] Gaiday A., Balash L., Tussupkaliyev A. (2022). The Role of High Concentrations of Homocysteine for the Development of Fetal Growth Restriction. Rev. Bras. Ginecol. Obstet..

[186] Rochtus A., Jansen K., Van Geet C., Freson K. (2015). Nutri-epigenomic Studies Related to Neural Tube Defects: Does Folate Affect Neural Tube Closure Via Changes in DNA Methylation?. Mini Rev. Med. Chem..

[187] Zaganjor I., Sekkarie A., Tsang B.L., Williams J., Razzaghi H., Mulinare J., Sniezek J.E., Cannon M.J., Rosenthal J. (2016). Describing the Prevalence of Neural Tube Defects Worldwide: A Systematic Literature Review. PLoS ONE.

[188] Blencowe H., Kancherla V., Moorthie S., Darlison M.W., Modell B. (2018). Estimates of global and regional prevalence of neural tube defects for 2015: A systematic analysis. Ann. N. Y. Acad. Sci..

[189] Khoshnood B., Loane M., de Walle H., Arriola L., Addor M.C., Barisic I., Beres J., Bianchi F., Dias C., Draper E. (2015). Long term trends in prevalence of neural tube defects in Europe: Population based study. BMJ.

[190] Shookhoff J.M., Gallicano G.I. (2010). A new perspective on neural tube defects: Folic acid and microRNA misexpression. Genesis.

[191] Beaudin A.E., Stover P.J. (2007). Folate-mediated one-carbon metabolism and neural tube defects: Balancing genome synthesis and gene expression. Birth Defects Res. C Embryo Today.

[192] Wang L., Shangguan S., Xin Y., Chang S., Wang Z., Lu X., Wu L., Niu B., Zhang T. (2017). Folate deficiency disturbs hsa-let-7 g level through methylation regulation in neural tube defects. J. Cell. Mol. Med..

[193] Moshtaghioon S., Elahi M., Ebrahim Soltani Z., Ahmadi E., Nabian M.H. (2025). MicroRNA regulation in neural tube defects: Insights into pathogenesis and potential therapeutic targets. Gene.

[194] Gurugubelli K.R., Ballambattu V.B. (2024). Perspectives on folate with special reference to epigenetics and neural tube defects. Reprod. Toxicol..

[195] Yoo S., Montazeri A., Bennett D., Bo Y., Chen P., Duthie S., Jensen N., Kaminga A., Lai J.S., Li X. (2026). Folate and global health umbrella review series, part 1: Methodological framework and syntheses on anaemia and neural tube defects. J. Glob. Health.

[196] Lavery A.M., Brender J.D., Zhao H., Sweeney A., Felkner M., Suarez L., Canfield M.A. (2014). Dietary intake of choline and neural tube defects in Mexican Americans. Birth Defects Res. A Clin. Mol. Teratol..

[197] Shaw G.M., Finnell R.H., Blom H.J., Carmichael S.L., Vollset S.E., Yang W., Ueland P.M. (2009). Choline and risk of neural tube defects in a folate-fortified population. Epidemiology.

[198] Shaw G.M., Carmichael S.L., Yang W., Selvin S., Schaffer D.M. (2004). Periconceptional dietary intake of choline and betaine and neural tube defects in offspring. Am. J. Epidemiol..

[199] Mills J.L., Fan R., Brody L.C., Liu A., Ueland P.M., Wang Y., Kirke P.N., Shane B., Molloy A.M. (2014). Maternal choline concentrations during pregnancy and choline-related genetic variants as risk factors for neural tube defects. Am. J. Clin. Nutr..

[200] Carmichael S.L., Yang W., Shaw G.M. (2010). Periconceptional nutrient intakes and risks of neural tube defects in California. Birth Defects Res. A Clin. Mol. Teratol..

[201] Wahbeh F., Manyama M. (2021). The role of Vitamin B12 and genetic risk factors in the etiology of neural tube defects: A systematic review. Int. J. Dev. Neurosci..

[202] Ray J.G., Blom H.J. (2003). Vitamin B12 insufficiency and the risk of fetal neural tube defects. QJM.

[203] Nie L., Liu X., Li X., Ren Z., Cheng X., Wu Y., Li Z., Liu J. (2025). Beyond Folate: The Emerging Role of Maternal Vitamin B12 in Neural Tube Development. Nutrients.

[204] Rubini E., Baijens I.M.M., Horanszky A., Schoenmakers S., Sinclair K.D., Zana M., Dinnyes A., Steegers-Theunissen R.P.M., Rousian M. (2021). Maternal One-Carbon Metabolism during the Periconceptional Period and Human Foetal Brain Growth: A Systematic Review. Genes.

[205] Derbyshire E., Maes M. (2023). The Role of Choline in Neurodevelopmental Disorders—A Narrative Review Focusing on ASC, ADHD and Dyslexia. Nutrients.

[206] Quadros E.V. (2023). Folate and Other B Vitamins in Brain Health and Disease. Nutrients.

[207] Bernhard W., Bockmann K.A., Minarski M., Wiechers C., Busch A., Bach D., Poets C.F., Franz A.R. (2024). Evidence and Perspectives for Choline Supplementation during Parenteral Nutrition—A Narrative Review. Nutrients.

[208] Yang B., Fritsche K.L., Beversdorf D.Q., Gu Z., Lee J.C., Folk W.R., Greenlief C.M., Sun G.Y. (2019). Yin-Yang Mechanisms Regulating Lipid Peroxidation of Docosahexaenoic Acid and Arachidonic Acid in the Central Nervous System. Front. Neurol..

[209] Zeisel S.H., Niculescu M.D. (2006). Perinatal choline influences brain structure and function. Nutr. Rev..

[210] Nguyen H.T., Oktayani P.P.I., Lee S.D., Huang L.C. (2025). Choline in pregnant women: A systematic review and meta-analysis. Nutr. Rev..

[211] Zhu J., Liu Y.H., He X.L., Kohlmeier M., Zhou L.L., Shen L.W., Yi X.X., Tang Q.Y., Cai W., Wang B. (2020). Dietary Choline Intake during Pregnancy and PEMT rs7946 Polymorphism on Risk of Preterm Birth: A Case-Control Study. Ann. Nutr. Metab..

[212] Wang H., Li J., Liu J., Leng J., Li W., Yu Z., Tam C.H.T., Hu G., Ma R.C.W., Fang Z. (2022). Interactions of CDKAL1 rs7747752 polymorphism and serum levels of L-carnitine and choline are related to increased risk of gestational diabetes mellitus. Genes Nutr..

[213] Ma S., Bo Y., Zhao X., Cao Y., Duan D., Dou W., Fu W., Zeng F., Lyu Q., Liu Y. (2022). One-carbon metabolism-related nutrients intake is associated with lower risk of preeclampsia in pregnant women: A matched case-control study. Nutr. Res..

[214] Huo X., Li J., Cao Y.F., Li S.N., Shao P., Leng J., Li W., Liu J., Yang K., Ma R.C.W. (2019). Trimethylamine N-Oxide Metabolites in Early Pregnancy and Risk of Gestational Diabetes: A Nested Case-Control Study. J. Clin. Endocrinol. Metab..

[215] Carmichael S.L., Yang W., Shaw G.M., The National Birth Defects Prevention Study (2013). Maternal dietary nutrient intake and risk of preterm delivery. Am. J. Perinatol..

[216] Hoffman M.C., Hunter S.J., D’Alessandro A., Christians U., Law A.J., Freedman R. (2024). Maternal Plasma Choline during Gestation and Small for Gestational Age Infants. Am. J. Perinatol..

[217] Nakanishi M., Funahashi N., Fukuoka H., Nammo T., Sato Y., Yoshihara H., Oishi H., Tanaka M., Yano T., Minoura S. (2021). Effects of maternal and fetal choline concentrations on the fetal growth and placental DNA methylation of 12 target genes related to fetal growth, adipogenesis, and energy metabolism. J. Obstet. Gynaecol. Res..

[218] Jiang X., Jones S., Andrew B.Y., Ganti A., Malysheva O.V., Giallourou N., Brannon P.M., Roberson M.S., Caudill M.A. (2014). Cho-line inadequacy impairs trophoblast function and vascularization in cultured human placental trophoblasts. J. Cell. Physiol..

[219] Jennings L., Basiri R. (2022). Amino Acids, B Vitamins, and Choline May Independently and Collaboratively Influence the Incidence and Core Symptoms of Autism Spectrum Disorder. Nutrients.

[220] Zwierz M., Suprunowicz M., Mrozek K., Pietruszkiewicz J., Oracz A.J., Konarzewska B., Waszkiewicz N. (2025). Vitamin B12 and Autism Spectrum Disorder: A Review of Current Evidence. Nutrients.

[221] Rush E.C., Katre P., Yajnik C.S. (2014). Vitamin B12: One carbon metabolism, fetal growth and programming for chronic disease. Eur. J. Clin. Nutr..

[222] Thornburg K.L., Valent A.M. (2024). Maternal Malnutrition and Elevated Disease Risk in Offspring. Nutrients.

[223] Socha M.W., Flis W., Wartega M. (2024). Epigenetic Genome Modifications during Pregnancy: The Impact of Essential Nutritional Supplements on DNA Methylation. Nutrients.

[224] Esfandiarei M., Bottiglieri T., Jadavji N.M. (2024). Modifying Levels of Maternal Dietary Folic Acid or Choline to Study the Impact of Deficiencies on Offspring Health Outcomes. J. Vis. Exp..

[225] Bernhard W., Poets C.F., Franz A.R. (2019). Choline and choline-related nutrients in regular and preterm infant growth. Eur. J. Nutr..

[226] Bekdash R.A. (2023). Methyl Donors, Epigenetic Alterations, and Brain Health: Understanding the Connection. Int. J. Mol. Sci..

[227] van Vliet M.M., Schoenmakers S., Gribnau J., Steegers-Theunissen R.P.M. (2024). The one-carbon metabolism as an underlying pathway for placental DNA methylation—A systematic review. Epigenetics.

[228] Eichenauer H., Ehlert U. (2023). The association between prenatal famine, DNA methylation and mental disorders: A systematic review and meta-analysis. Clin. Epigenet..

[229] Herrera-Cuenca M., Yepez Garcia M.C., Cortes Sanabria L.Y., Hernandez P., Ramirez G., Vasquez M., Sifontes Y., Gomez G., Liria-Dominguez M.R., Rigotti A. (2024). Inadequate Intake of Choline and Essential Fatty Acids in Latin American Childbearing-Age Women as a Regional Pre-Conceptional Disadvantage: ELANS Results. Nutrients.

[230] Iglesias-Vazquez L., Suliburska J., Kocylowski R., Bakinowska E., Arija V. (2023). Nutrient Intake among Pregnant Women in Spain and Poland: A Comparative Analysis. Nutrients.

[231] Obeid R., Schon C., Wilhelm M., Pietrzik K., Pilz S. (2018). The effectiveness of daily supplementation with 400 or 800 microg/day folate in reaching protective red blood folate concentrations in non-pregnant women: A randomized trial. Eur. J. Nutr..

[232] Thurston L., Borman B., Bower C. (2023). Mandatory fortification with folic acid for the prevention of neural tube defects: A case study of Australia and New Zealand. Childs Nerv. Syst..

[233] Wilson R.D., O’Connor D.L. (2021). Maternal folic acid and multivitamin supplementation: International clinical evidence with considerations for the prevention of folate-sensitive birth defects. Prev. Med. Rep..

[234] FSA Fortification of Flour with Folic Acid. https://www.food.gov.uk/safety-hygiene/folic-acid?utm_source.

[235] Chen M.Y., Rose C.E., Qi Y.P., Williams J.L., Yeung L.F., Berry R.J., Hao L., Cannon M.J., Crider K.S. (2019). Defining the plasma folate concentration associated with the red blood cell folate concentration threshold for optimal neural tube defects prevention: A population-based, randomized trial of folic acid supplementation. Am. J. Clin. Nutr..

[236] Thompson M.D., Cole D.E., Ray J.G. (2009). Vitamin B-12 and neural tube defects: The Canadian experience. Am. J. Clin. Nutr..

[237] Gallina A.L., Otay S., de Frutos-Lucas J., Buso M., Moral Martinez P., Cashman K.D., Kiely M.E., Astley S. (2025). Hidden hunger in Europe: A review on determinants, fragmented policy responses, and implementation barriers. Front. Nutr..

[238] DHSC Fortified Foods: Guidance to Compliance on European Regulation (EC) No. 1925/2006 on the Addition of Vitamins and Minerals and Certain Other Substances to Food. https://www.gov.uk/government/publications/fortified-foods-guidance-to-compliance-with-european-regulation-ec-no-1925-2006-on-the-addition-of-vitamins-and-minerals-and-certain-other-substances-to-food/fortified-foods-guidance-to-compliance-on-european-regulation-ec-no-19252006-on-the-addition-of-vitamins-and-minerals-and-certain-other-substance?.

[239] Neufeld L.M., Ho E., Obeid R., Tzoulis C., Green M., Huber L.G., Stout M., Griffiths J.C. (2023). Advancing nutrition science to meet evolving global health needs. Eur. J. Nutr..

[240] Obeid R., Karlsson T. (2023). Choline—A scoping review for Nordic Nutrition Recommendations 2023. Food Nutr. Res..

[241] NNR (2023). Nordic Nutrition Recommendations. https://pub.norden.org/nord2023-003/index.html.

[242] Yan J., Jiang X., West A.A., Perry C.A., Malysheva O.V., Devapatla S., Pressman E., Vermeylen F., Stabler S.P., Allen R.H. (2012). Maternal choline intake modulates maternal and fetal biomarkers of choline metabolism in humans. Am. J. Clin. Nutr..

[243] IOM (1998). Dietary Reference Intakes for Thiamin, Riboflavin, Niacin, Vitamin B_6_, Folate, Vitamin B_12_, Pantothenic Acid, Biotin, and Choline; A Report of the Standing Committee on the Scientific Evaluation of Dietary Reference Intakes and Its Panel on Folate, Other B Vitamins, and Choline and Subcommittee on Upper Reference Levels of Nutrients, Food and Nutrition Board, Institute of Medicine. https://www.nationalacademies.org/publications/6015.

[244] Adams J.B., Kirby J.K., Sorensen J.C., Pollard E.L., Audhya T. (2022). Evidence based recommendations for an optimal prenatal supplement for women in the US: Vitamins and related nutrients. Matern Health Neonatol. Perinatol..

[245] Masih S.P., Plumptre L., Ly A., Berger H., Lausman A.Y., Croxford R., Kim Y.I., O’Connor D.L. (2015). Pregnant Canadian Women Achieve Recommended Intakes of One-Carbon Nutrients through Prenatal Supplementation but the Supplement Composition, Including Choline, Requires Reconsideration. J. Nutr..

[246] Niculescu M.D., Zeisel S.H. (2002). Diet, methyl donors and DNA methylation: Interactions between dietary folate, methionine and choline. J. Nutr..

[247] Mahfouz R., Akiki M.T., Ndayra V., El Khoury R., Chawi M., Hatem M., Hanna-Wakim L., Sacre Y., Hoteit M. (2024). Energy, Macronutrients and Micronutrients Intake Among Pregnant Women in Lebanon: Findings from the Updated Lebanese National Food Consumption Survey (LEBANON-FCS). Nutrients.

[248] Hart K.H., Hill A.J., Gonzalez J.T., de la Hunty A., Gallagher A.M., Stanner S.A. (2025). Diet in Pregnancy: A Review of Current Challenges and Recommendations. A British Nutrition Foundation Briefing Paper. Nutr. Bull..

[249] Kadam I., Dalloul M., Hausser J., Vaday D., Gilboa E., Wang L., Hittelman J., Hoepner L., Fordjour L., Chitamanni P. (2024). Role of one-carbon nutrient intake and diabetes during pregnancy in children’s growth and neurodevelopment: A 2-year follow-up study of a prospective cohort. Clin. Nutr..

[250] Chungchunlam S.M.S., Moughan P.J. (2024). Comparative bioavailability of vitamins in human foods sourced from animals and plants. Crit. Rev. Food Sci. Nutr..

[251] Eichholzer M., Tonz O., Zimmermann R. (2006). Folic acid: A public-health challenge. Lancet.

[252] Savard C., Plante A.S., Carbonneau E., Gagnon C., Robitaille J., Lamarche B., Lemieux S., Morisset A.S. (2020). Do pregnant women eat healthier than non-pregnant women of childbearing age?. Int. J. Food Sci. Nutr..

[253] Zerback T., Koeder C., Weder S., Sputtek A., Eckert G.P., Keller M. (2025). Assessment of vitamin A, vitamin B_2_, vitamin B_12_, vitamin K, folate, and choline status following 4 months of multinutrient supplementation in healthy vegans: A randomised, double-blind, placebo-controlled trial. Eur. J. Nutr..

[254] Goldman D., Nagra M. (2025). Choline adequacy and health outcomes in vegetarian and vegan diets. Acad. Nutr. Diet..

[255] Imbard A., Benoist J.F., Blom H.J. (2013). Neural tube defects, folic acid and methylation. Int. J. Environ. Res. Public Health.

[256] Wu B.T., Innis S.M., Mulder K.A., Dyer R.A., King D.J. (2013). Low plasma vitamin B-12 is associated with a lower pregnancy-associated rise in plasma free choline in Canadian pregnant women and lower postnatal growth rates in their male infants. Am. J. Clin. Nutr..

[257] Bodnaruc A.M., Khan H., Shaver N., Bennett A., Wong Y.L., Gracey C., Ly V., Shea B., Little J., Brouwers M. (2025). Reliability and reproducibility of systematic reviews informing the 2020-2025 Dietary Guidelines for Americans: A pilot study. Am. J. Clin. Nutr..

[258] Moore C.J., Perreault M., Mottola M.F., Atkinson S.A. (2020). Diet in Early Pregnancy: Focus on Folate, Vitamin B12, Vitamin D, and Choline. Can. J. Diet. Pract. Res..

[259] Cho C.E., Aardema N.D.J., Bunnell M.L., Larson D.P., Aguilar S.S., Bergeson J.R., Malysheva O.V., Caudill M.A., Lefevre M. (2020). Effect of Choline Forms and Gut Microbiota Composition on Trimethylamine-N-Oxide Response in Healthy Men. Nutrients.

[260] Fardous A.M., Heydari A.R. (2023). Uncovering the Hidden Dangers and Molecular Mechanisms of Excess Folate: A Narrative Review. Nutrients.

[261] Colapinto C.K., O’Connor D.L., Sampson M., Williams B., Tremblay M.S. (2016). Systematic review of adverse health outcomes associated with high serum or red blood cell folate concentrations. J. Public Health.

[262] Khalighi Sikaroudi M., Soltani S., Kolahdouz-Mohammadi R., Imanifard R., Abdollahi S., Shahinfar H., Mohammadi Farsani G. (2024). The association between dietary folate intake and risk of colorectal cancer incidence: A systematic review and dose-response meta-analysis of cohort studies. Heliyon.

[263] Thabet R.H., Alessa R.E.M., Al-Smadi Z.K.K., Alshatnawi B.S.G., Amayreh B.M.I., Al-Dwaaghreh R.B.A., Salah S.K.A. (2024). Folic acid: Friend or foe in cancer therapy. J. Int. Med. Res..

[264] Lamers Y., MacFarlane A.J., O’Connor D.L., Fontaine-Bisson B. (2018). Periconceptional intake of folic acid among low-risk women in Canada: Summary of a workshop aiming to align prenatal folic acid supplement composition with current expert guidelines. Am. J. Clin. Nutr..

[265] Turck D., Bohn T., Castenmiller J., de Henauw S., Hirsch-Ernst K.I., Knutsen H.K., Maciuk A., Mangelsdorf I., McArdle H.J., EFSA (2023). Scientific opinion on the tolerable upper intake level for folate. EFSA J..

[266] Derbyshire E. (2019). Could we be overlooking a potential choline crisis in the United Kingdom?. BMJ Nutr. Prev. Health.

[267] Almekkawi A.K., AlJardali M.W., Daadaa H.M., Lane A.L., Worner A.R., Karim M.A., Scheck A.C., Frye R.E. (2022). Folate Pathway Gene Single Nucleotide Polymorphisms and Neural Tube Defects: A Systematic Review and Meta-Analysis. J. Pers. Med..

[268] Chmurzynska A., Seremak-Mrozikiewicz A., Malinowska A.M., Rozycka A., Radziejewska A., KurzawiNska G., Barlik M., Wolski H., Drews K. (2020). Associations between folate and choline intake, homocysteine metabolism, and genetic polymorphism of MTHFR, BHMT and PEMT in healthy pregnant Polish women. Nutr. Diet..

[269] Amenyah S.D., Hughes C.F., Ward M., Rosborough S., Deane J., Thursby S.J., Walsh C.P., Kok D.E., Strain J.J., McNulty H. (2020). Influence of nutrients involved in one-carbon metabolism on DNA methylation in adults-a systematic review and meta-analysis. Nutr. Rev..

[270] Bekdash R.A. (2021). Early Life Nutrition and Mental Health: The Role of DNA Methylation. Nutrients.

